# Divergent assembly patterns of core and non-core microbial taxa under low environmental heterogeneity in the surface waters of the Xisha Islands

**DOI:** 10.1128/spectrum.02178-26

**Published:** 2026-08-14

**Authors:** Zhixuan Deng, Renjun Zhou, Dongwei Hou, Shenzheng Zeng, Shaoping Weng, Jianguo He, Zhijian Huang

**Affiliations:** 1 State Key Laboratory of Biocontrol, Southern Marine Sciences and Engineering Guangdong Laboratory (Zhuhai), School of Agriculture and Biotechnology, School of Marine Sciences, Sun Yat-sen University 626366, Shenzhen, Guangzhou, China; 2 School of Life Sciences, Sun Yat-sen University 26469, Guangzhou, China; Cleveland Clinic Lerner Research Institute, Cleveland, Ohio, USA

**Keywords:** marine microbial community, surface water, core microbiota, community assembly, Xisha Islands

## Abstract

Microbial communities play essential roles in marine biogeochemical cycling, yet the respective roles of environmental and spatial drivers of community assembly remain incompletely understood, particularly under low environmental heterogeneity and across distinct microbial subcommunities. We investigated microbial communities from 39 surface-water samples collected across the Xisha Islands (South China Sea) by distinguishing core and non-core taxa to assess associations of environmental gradients and geographic distance with community composition, assembly, and predicted functional potential. Physicochemical conditions varied narrowly (temperature 29.5°C–31.5°C; salinity 30.47%–30.93%, pH 7.85–8.22), reflecting low environmental heterogeneity. Core taxa comprised 1.15% of richness and contributed >50% of total abundance, whereas non-core taxa accounted for 98.85% of richness and disproportionately structured the co-occurrence network. Environmental variables (particularly temperature and pH) together with geographic distance were significantly associated with community composition, with variation partitioning revealing a relatively greater spatial component in non-core communities, consistent with their stronger spatial structuring (distance-decay slopes: −0.118 vs −0.061 for core taxa). Assembly analyses revealed contrasting patterns: core-community comparisons were classified as dispersal limitation, whereas non-core comparisons were primarily associated with homogeneous selection (46.96%) and dispersal limitation (45.21%). Predicted community functional structure was primarily associated with temperature, pH, and redox conditions. These findings suggest that environmental gradients and geographic distance are associated with microbial community composition, assembly, and predicted functional potential in marine environments with low environmental heterogeneity, and core and non-core taxa exhibit contrasting assembly patterns. This study highlights the importance of considering microbial subcommunities when investigating community assembly in marine ecosystems.

**IMPORTANCE:**

Microbial communities play fundamental roles in marine biogeochemical cycling. However, most understanding of microbial community assembly derives from systems with strong environmental gradients or large spatial scales, where pronounced heterogeneity may obscure subtle ecological processes. This bias limits our understanding of microbial community assembly and functional potential in relatively stable marine environments, where subtle environmental and spatial gradients may still exert important influences. In addition, whether microbial groups with contrasting occurrence patterns respond differently to these processes remains insufficiently evaluated. This study shows that, despite the relatively low environmental heterogeneity of tropical marine surface waters in the Xisha Islands, environmental gradients (particularly temperature and pH) and geographic distance remain associated with microbial community composition, assembly, and predicted functional potential. In addition, core and non-core taxa exhibited contrasting assembly patterns. Our findings provide a framework for understanding microbial community assembly by considering core and non-core subcommunities separately.

## INTRODUCTION

Marine microbial communities harbor immense taxonomic diversity and play fundamental roles in global biogeochemical cycles and ecosystem functioning due to their high abundance and metabolic activity (1, 2). Microbial community composition is highly sensitive to environmental change and can rapidly respond to variations in environmental conditions, making them important indicators of ecosystem dynamics (3–6). Environmental variation is also closely linked to both the taxonomic structure and functional potential of microbial communities, thereby influencing ecosystem processes (7–10). A central goal in microbial ecology is to understand the mechanisms underlying community assembly and the processes that generate observed patterns of diversity and composition (11, 12). In this context, community assembly is generally considered to be governed by a combination of deterministic processes (e.g., environmental filtering) and stochastic processes (e.g., dispersal and ecological drift) (13, 14). However, much of our current understanding of marine microbial community assembly has been derived from systems characterized by pronounced environmental gradients or broad spatial scales, such as estuarine ecosystems (15), coastal upwelling regions (16), and global ocean surveys (17), where strong environmental heterogeneity may obscure the ecological influences of more subtle environmental and spatial gradients. Consequently, how environmental processes (i.e., ecological processes related to environmental conditions) and spatial processes (i.e., ecological processes related to spatial structure and geographic distance) contribute to microbial community assembly in marine environments with relatively low environmental heterogeneity remains insufficiently understood.

Microbial communities often exhibit uneven abundance distributions, characterized by a small proportion of highly abundant taxa and a large number of low-abundance taxa (18, 19). The core microbiota generally refers to taxa that occur with high frequency and relatively stable abundance within a given ecological scale (20). Core taxa are often considered to play key roles in maintaining community stability, whereas non-core taxa contribute to the complexity of microbial interactions (21–23). Importantly, accumulating evidence suggests that microbial taxa with different occurrence frequencies and abundance levels may exhibit distinct assembly patterns and ecological responses to environmental and spatial processes. Previous studies have suggested that core taxa are often more closely associated with stochastic processes, whereas non-core or low-abundance taxa may exhibit stronger associations with deterministic environmental filtering. However, different ecosystems have exhibited contrasting patterns regarding the relative contributions of deterministic and stochastic processes to the assembly of core and non-core taxa (24–26). Therefore, explicitly distinguishing between core and non-core groups is essential for a more comprehensive understanding of microbial community assembly and its underlying drivers (27).

Marine island ecosystems and their surrounding waters provide valuable natural settings for investigating biodiversity patterns and ecological processes and have attracted extensive scientific attention (28–30). The Xisha Islands, a group of tropical oceanic islands in the South China Sea, provide a suitable regional system for investigating microbial community assembly under relatively homogeneous environmental conditions (31). Despite their ecological importance, previous studies in this region have mainly focused on seabird communities and sediment-associated microorganisms (32, 33). However, microbial communities in the surrounding surface waters remain poorly characterized, particularly with respect to the roles of environmental and spatial factors in shaping community composition and assembly. Moreover, a more detailed understanding of how different microbial groups respond to these factors is still lacking, as is the extent to which environmental variation is associated with microbial functional potential.

To address these gaps, we investigated microbial communities in the surface waters surrounding the Xisha Islands by integrating analyses of environmental and spatial variables, community composition, and predicted functional potential. Our specific objectives were to (i) characterize the environmental and geographic features of surface waters across the study sites; (ii) describe the composition and diversity of microbial communities, with explicit differentiation between core and non-core taxa; (iii) evaluate how environmental and spatial factors influence microbial groups with contrasting occurrence patterns; (iv) assess the relative roles of deterministic and spatial processes in community assembly; and (v) examine the relationships between environmental variables and microbial functional potential. Collectively, this study provides a framework for understanding how environmental and spatial factors are associated with microbial community structure, assembly patterns, and functional potential in tropical marine surface waters and offers insights for the management of microbial dynamics in island ecosystems.

## MATERIALS AND METHODS

### Sample collection and physicochemical analysis

A total of 39 seawater samples were collected from 13 sites (named BJDA, ZSDA, ZSDB, DDDA, DDDB, QFDA, YLHA, LYJA, HGJA, HGJB, PSYA, LHJA, and ZJDA, respectively) with three replicates per site, in the Xisha Islands, South China Sea (15.803°–17.100° N, 111.216°–112.755° E), from 15 April to 15 May 2019. The sampling sites were distributed across the Xisha Islands to represent the regional spatial extent of the surface-water system and capture naturally occurring environmental variation among sampling locations. All samples were collected from a research vessel in the surface waters surrounding the islands. For each sample, 2.0 L of seawater was collected from a depth of 5–10 cm below the surface using sterilized plastic bottles. After collection, 1 L of the seawater was filtered with a 0.22 μm polyethersulfone membrane filter (Supor-200, Pall, New York, USA) using a vacuum pump. The membranes containing microbial biomass were stored at −80°C until DNA extraction (7, 34).

Temperature (T), dissolved oxygen (DO), oxidation-reduction potential (ORP), pH, and salinity (SAL) were measured *in situ* using a Yellow Springs Instrument (YSI) handheld multiparameter instrument (Model YSI 380, YSI Incorporated, USA). For physicochemical analyses, 100 mL of each sample was collected in sterile bottles. Samples used for dissolved nutrient analyses (ammonia nitrogen [NH_4_^+^-N], nitrite nitrogen [NO_2_^−^-N], nitrate nitrogen [NO_3_^−^-N], and orthophosphate [PO_4_^3−^-P]) were filtered through a 0.22 μm membrane immediately after collection to remove most microbial cells before storage at 4°C until analysis. Samples for total nitrogen (TN) and total phosphate (TP) analyses were stored without filtration according to the analytical protocol. Nutrient concentrations were determined using an automatic discrete analyzer (CleverChem 380, DeChem-tech, Germany) (7).

### DNA extraction and 16S rRNA gene sequencing

Total genomic DNA was extracted using the Water DNA Kit (MinkaGene, China) according to the manufacturer’s instructions. DNA concentration and purity were determined using a Nano Vue Plus spectrophotometer (GE Healthcare, USA). For 16S rRNA gene amplicon sequencing, the V4 hypervariable region was amplified using primers 515F (5′-GTGCCAGCMGCCGCGGTAA-3′) and 806R (5′-GGACTACHVGGGTWTCTAAT-3′; 35). Sequencing libraries were constructed using a TruSeq DNA PCR-Free Sample Preparation Kit (Illumina, USA), and index codes were added. The libraries were sequenced on an Illumina HiSeq 2500 platform (Illumina, USA) at Novogene Biotech Co., Ltd (Beijing, China). Raw sequencing data have been deposited in the NCBI Sequence Read Archive under accession number PRJNA1460329.

### Sequencing data processing

Sequence data were processed using DADA2 and quantitative insights into microbial ecology version 2 (QIIME2) (36). Trimming, merging, quality filtering, and amplicon sequence variant (ASVs) inference were performed using DADA2. Taxonomic classification was conducted against the SILVA database (version 138) (37). Alpha and beta diversity metrics were calculated using QIIME 2 (38). Functional profiles were predicted from 16S rRNA gene sequences using phylogenetic investigation of communities by reconstruction of unobserved states (PICRUSt2) (39). Predicted functional pathways were annotated against the Kyoto Encyclopedia of Genes and Genomes (KEGG) database at level 3 ortholog groups (KOs). Prediction accuracy was evaluated using the nearest sequenced taxon index (NSTI). Currently, there is no universal standard for defining core and non-core taxa. ASVs detected in all samples and with a relative abundance ≥0.2% in more than 80% of samples were defined as core taxa, while all remaining ASVs were classified as non-core taxa (20, 40, 41).

### Construction of co-occurrence network

Co-occurrence networks were constructed based on ASVs from all samples using the “igraph” and “psych” packages in R and visualized in Gephi software (42). To reduce complexity, only ASVs present in more than 80% of samples were retained. Pairwise Spearman’s correlations were calculated between ASVs, and correlations with |*r*| > 0.7 and *P* < 0.01 were considered statistically significant (43).

### Estimation of ecological processes

Community assembly processes were evaluated using a combination of phylogenetic and abundance-based null models following Stegen et al. (44, 45). Phylogenetic turnover was quantified using the β-mean nearest taxon distance calculated with the “picante” package in R. The β-nearest taxon index (βNTI) was used to infer ecological processes. Values of |βNTI| > 2 indicate dominance of deterministic processes, whereas |βNTI| < 2 suggest stochastic processes. To further partition stochastic processes, the Bray-Curtis-based Raup-Crick metric (RC_bray_) was calculated. RC_bray_ > 0.95, RC_bray_ < −0.95, and |RC_bray_| ≤ 0.95 indicate dispersal limitation, homogenizing dispersal, and drift, respectively (46).

### Statistical analysis

Venn diagrams were generated to identify shared and unique ASVs among sites using the R package “Venn Diagram” (47). To quantify the independent and shared contributions of environmental and spatial variables to microbial community variation, variation partitioning analysis was performed using the vegan package. Geographic coordinates (longitude and latitude) were used as spatial variables, and physicochemical parameters were included as environmental variables. Variable importance was evaluated as the percentage increase in mean squared error (%IncMSE) after permutation of each predictor, with higher %IncMSE values indicating greater importance for model prediction. Variable importance scores of environmental variables and geographic distance for predicting Bray-Curtis dissimilarity of microbial communities were determined using random forest analysis (48). Distance-decay relationships were assessed by regressing Bray-Curtis similarity against geographic distance among samples (49). Mantel test (vegan package in R) was used to examine the correlations between Euclidean distance of seawater physicochemical factors (geographic distance) and Bray-Curtis dissimilarity of microbial community (50). The relationships between the relative abundance of predicted functional profiles (relative abundance of ASVs and alpha diversity of microbial community) and the environmental variables were determined using Spearman rank correlation, with *P*-values adjusted using the false discovery rate method (51).

## RESULTS

### Environmental and geographic characteristics of surface waters in the Xisha Islands

**Fig 1:**
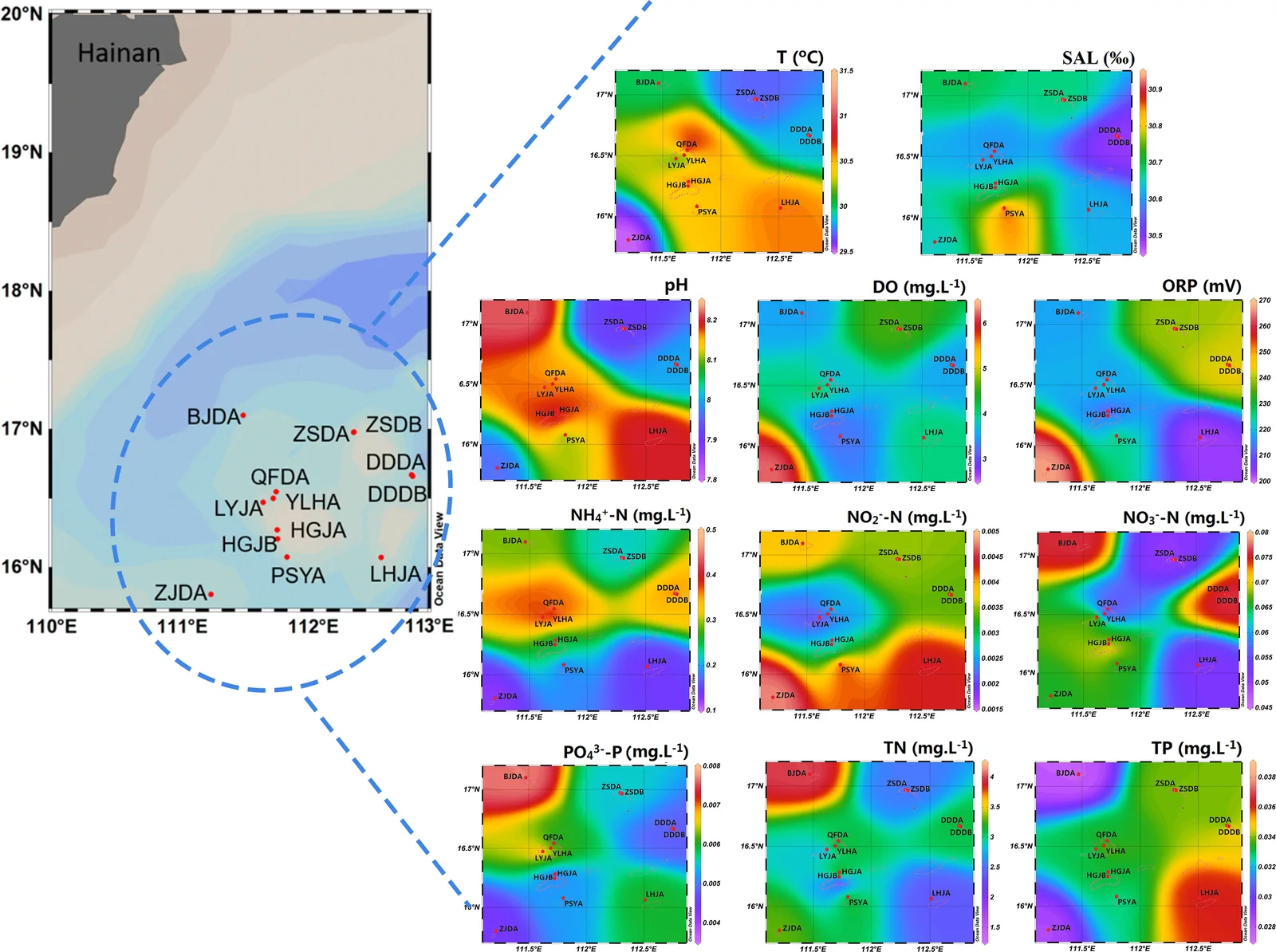
Environmental and geographic characteristics of surface waters in the Xisha Islands. Sampling sites were distributed across the regional surface waters of the Xisha Islands, representing the spatial extent of the study area. Physicochemical parameters of the surface waters of the Xisha Islands. The heatmap showing the variation in environmental parameters, with colors representing their values.

The geographic coordinates of all sampling sites are provided in Table S1. The mean seawater T was 30.1°C ± 0.6°C, SAL was 30.64% ± 0.11%, and pH was 8.09 ± 0.12. ORP was 228.1 ± 18.3 mV, and DO was 3.99 ± 0.85 mg L⁻¹ (Fig. 1; Tables S1 and S2).

Nutrient concentrations also exhibited relatively limited variation (Fig. 1; Table S1). NH₄^+^-N was 0.0268 ± 0.0101 mg L^−1^, NO_2_^−^-N was 0.0034 ± 0.0009 mg L^−1^, and NO_3_^−^-N was 0.0642 ± 0.0114 mg L^−1^. TN was 0.2127 ± 0.0596 mg L^−1^, PO_4_^3−^-P was 0.0056 ± 0.0012 mg L^−1^, and TP was 0.0329 ± 0.0029 mg L^−1^. Overall, physicochemical parameters showed relatively narrow variation across sites. These results indicate that physicochemical conditions in the surface waters of the Xisha Islands were relatively stable across the sampling sites.

### Composition, diversity, and co-occurrence network characteristics of core and non-core microbial taxa

**Fig 2:**
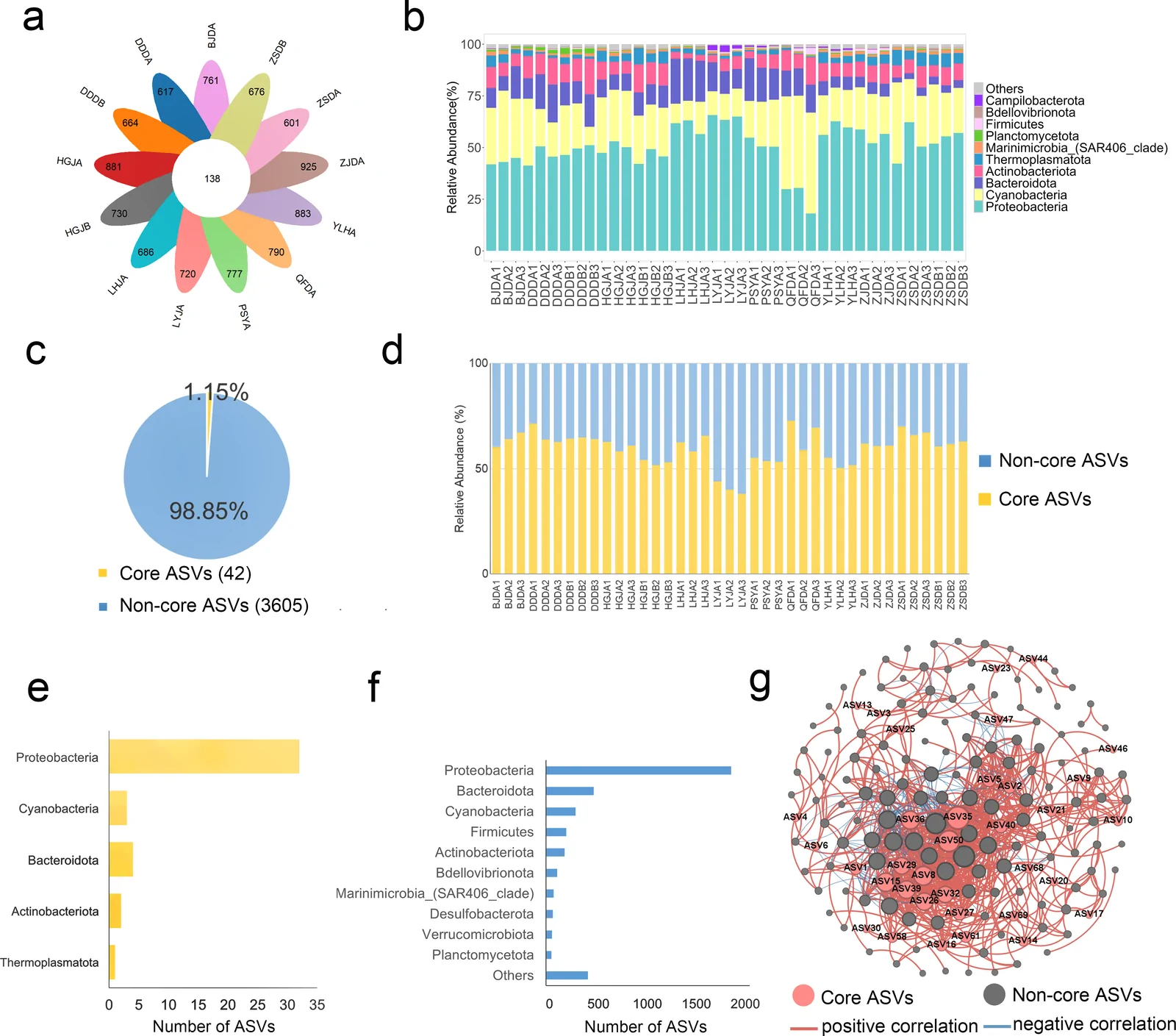
Composition and diversity of microbial communities in the surface waters of the Xisha Islands. (**a**) The unique and shared ASVs among the 13 sampling sites. (**b**) The 10 most abundant bacterial phyla in water. (**c**) Percentage of core and non-core taxa. (**d**) Relative abundance of core and non-core taxa. (**e**) Taxonomic composition of core taxa at the phylum level. (**f**) Taxonomic composition of non-core taxa at the phylum level. (**g**) Co-occurrence network analysis of microbial relationships. The network is based on pairwise Spearman’s correlations; red edges represent positive correlations, and blue edges represent negative correlations. Node size indicates the degree. Red nodes represent core ASVs, and gray nodes represent non-core ASVs.

A total of 3,647 bacterial ASVs were detected from 39 seawater samples. Venn diagram analysis identified 138 ASVs shared among the 13 sampling sites (Fig. 2a). The dominant phyla across all samples were Proteobacteria, Cyanobacteria, Bacteroidetes, Actinobacteria, Thermoplasmatota, and Marinimicrobia (SAR406 clade; Fig. 2b). At the genus level, the communities were mainly composed of Synechococcus CC9902, Prochlorococcus MIT9313, SAR86 clade, unclassified Rhodobacteraceae, and Candidatus *Actinomarina* (Fig. S1). Alpha diversity varied among sites, with richness ranging from 367 ± 14 in ZSDA to 540 ± 54 in YLHA, and Shannon index ranging from 2.97 ± 0.25 in QFDA to 4.22 ± 0.12 in YLHA (Table S3).

Core taxa represented a very small fraction of total community richness, accounting for only 1.15% (42 ASVs), whereas non-core taxa comprised the vast majority (98.85%, 3,605 ASVs; Fig. 2c). Despite their low richness, core taxa contributed more than 50% of the total relative abundance in most samples, with the exception of one sample from the LYJA site (Fig. 2d). Core taxa were predominantly affiliated with Proteobacteria (32), which accounted for the largest proportion, while Cyanobacteria (3), Bacteroidota (4), Actinobacteriota (2), and Thermoplasmatota (1) were present at lower relative abundances (Fig. 2e). In contrast, non-core taxa included not only Proteobacteria (1,817), Bacteroidota (467), and Cyanobacteria (290) but also a wider range of additional lineages, such as Firmicutes (198), Marinimicrobia (SAR406 clade; 75), and the predatory phylum Bdellovibrionota (110; Fig. 2f).

Network analysis identified 168 ASVs in the co-occurrence network, comprising 888 edges, with an average node degree of 10.509 and a clustering coefficient of 0.585. Positive correlations accounted for 88.15% of all edges. Among the network nodes, 37 (21.29%) were classified as core taxa, whereas 131 (78.71%) belonged to non-core taxa (Fig. 2g; Table S4). These results indicate that although core taxa dominate community abundance, non-core taxa contribute disproportionately to network structure.

### Environmental and spatial correlates of microbial communities across core and non-core taxa

Correlation analysis revealed that several environmental and geographic variables were significantly associated with microbial alpha diversity and community composition (Fig. S2a through e). T was negatively correlated with the Shannon index (*P* < 0.05), whereas NO_2_^−^-N was negatively correlated with richness and Chao1 indices (both *P* < 0.05). In contrast, longitude and latitude were positively correlated with both Shannon (*P* < 0.01) and Simpson indices (*P* < 0.001), indicating that spatial variation was linked to variation in community diversity. At the taxonomic level, T, pH, DO, and ORP were the primary factors associated with the relative abundances of both core and non-core taxa, exhibiting broadly similar correlation patterns (Fig. S2b and c). Most taxa associated with T and pH exhibited negative correlations, whereas those associated with DO and ORP were predominantly positively correlated. Longitude and latitude were additionally correlated with a large number of non-core taxa, particularly through positive associations (Fig. S2b through e). Overall, these results suggest that several key environmental and geographic variables were associated with microbial diversity and community composition despite the relatively low environmental heterogeneity.

**Fig 3:**
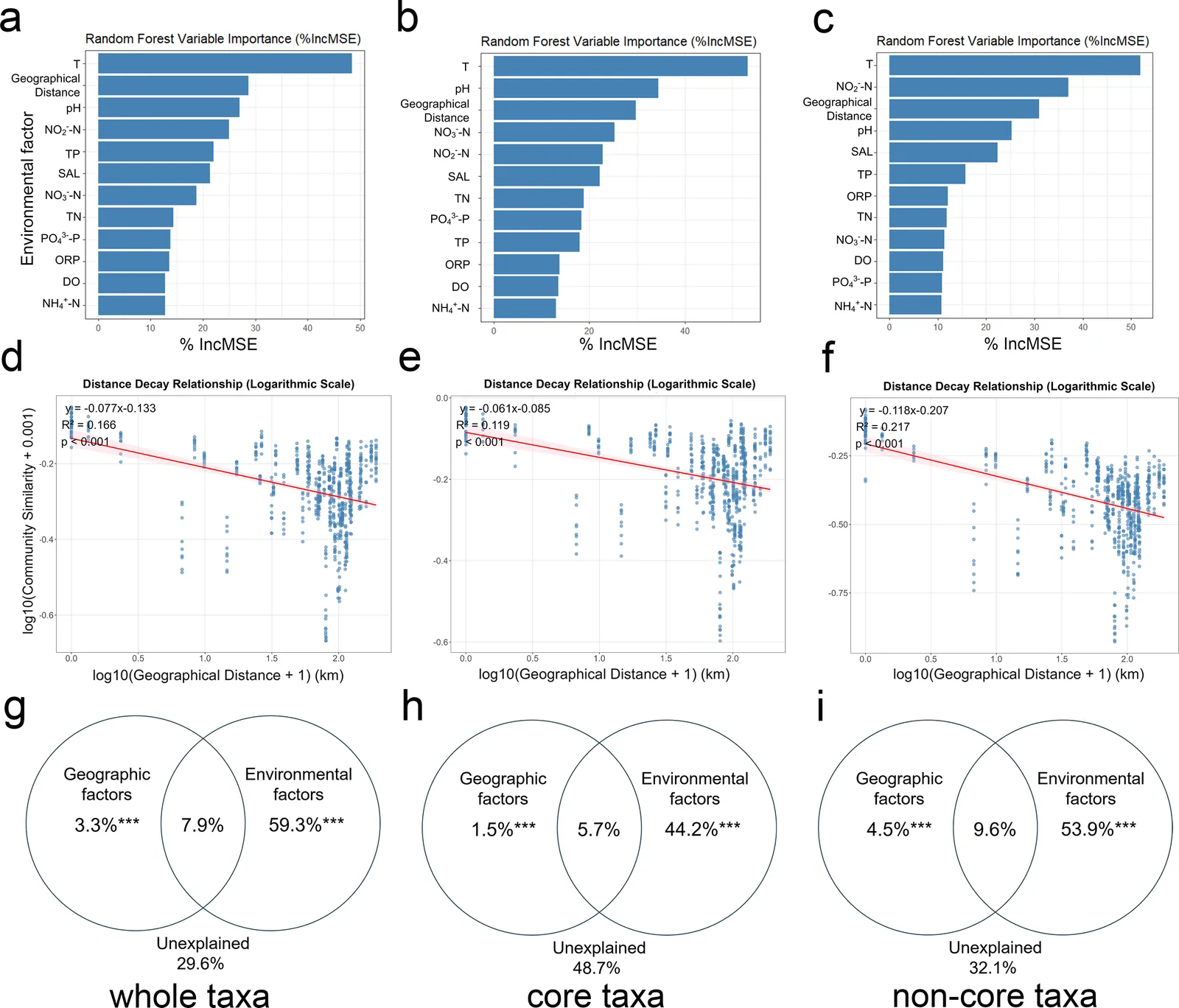
Environmental and geographic drivers of microbial community. Random forest analysis showing the importance of environmental variables and geographic distance in predicting Bray-Curtis community dissimilarity for the (**a**) whole community, (**b**) core taxa, and (**c**) non-core taxa. Variable importance is expressed as the percentage increase in mean squared error (%IncMSE), with larger values indicating a greater contribution to model performance. Distance-decay curves of similarity for the (**d**) whole community, (**e**) core taxa, and (**f**) non-core taxa. The red line represents the fitted linear regression with *R^−^*^2^ and *P* values labeled. Variation partitioning analysis of the effects of geographic factors and environmental factors the (**g**) whole community, (**h**) core taxa, and (**i**) non-core taxa bacterial structure. ***P < 0.01.

Random forest analysis showed that T had the highest variable importance across the whole, core, and non-core microbial communities (Fig. 3a through c). pH and geographic distance also showed consistently high variable importance across all three community types. In the whole community, T, geographic distance, and pH ranked as the top three variables, followed by NO_2_^−^-N and TP (Fig. 3a). For the core taxa, T and pH showed relatively high variable importance, while geographic distance and NO_3_^−^-N were also important contributors (Fig. 3b). In contrast, in the non-core taxa, NO_2_^−^-N and geographic distance ranked immediately after T, followed by pH and SAL (Fig. 3c). Mantel test results were broadly consistent with these patterns (Tables S5 through S7). The whole community showed a significant correlation with combined physicochemical and geographic factors (*r* = 0.231, *P* = 0.003), with T (*r* = 0.477, *P* = 0.001) and pH (*r* = 0.371, *P* = 0.001) showing the strongest associations. Similarly, T and pH were the strongest correlates of the core community (Table S6), whereas longitude, T, and NO_2_^−^-N showed the strongest associations with the non-core community (Table S7).

Distance-decay analysis showed that Bray-Curtis similarity decreased significantly with increasing geographic distance across all three community types (*P* < 0.001; Fig. 3d through f). The steeper slope and higher explanatory power observed for the non-core taxa (−0.118, *R*^2^ = 0.217) suggest a stronger spatial signal compared with the core taxa (−0.061, *R*^2^ = 0.119). Consistently, variation partitioning analysis showed that environmental variables explained the largest proportion of community variation across all community types, whereas the non-core community (4.5%) exhibited a relatively greater spatial component than the core community (1.5%; Fig. 3g through i), further supporting the stronger geographic signal observed for non-core taxa. Overall, temperature consistently showed the highest variable importance across community types, whereas geographic distance ranked among the most important variables in the non-core community.

### Contrasting assembly processes between core and non-core microbial communities

**Fig 4:**
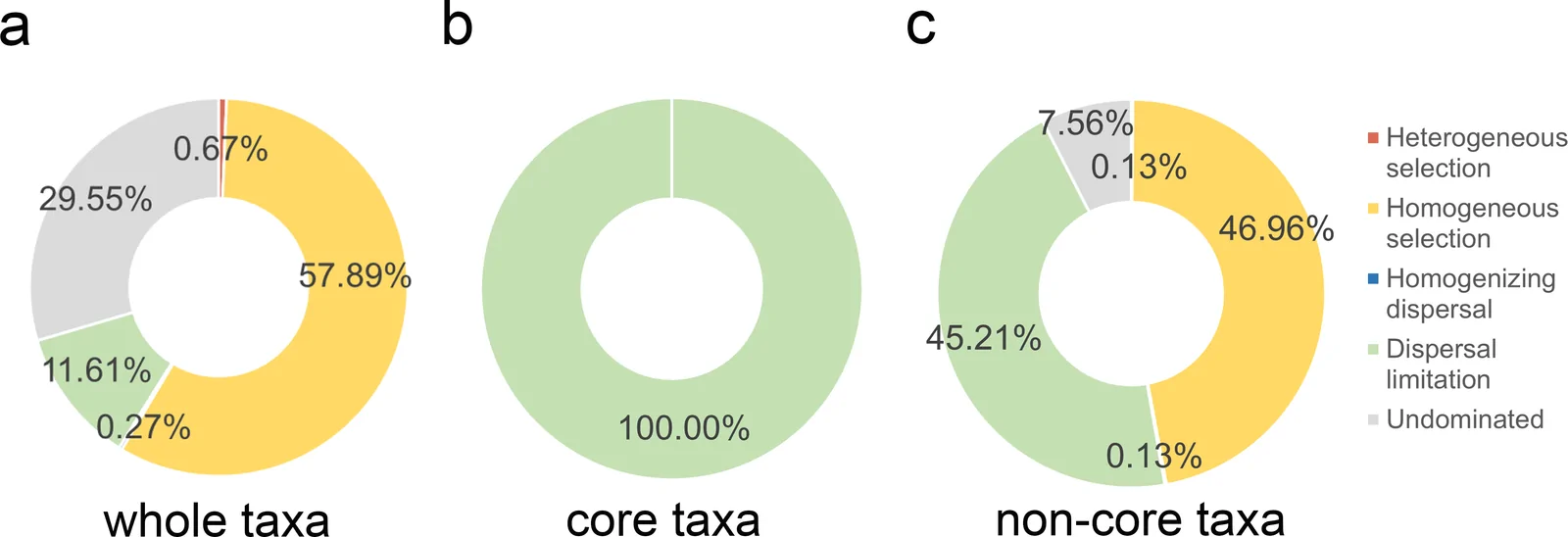
The contribution of ecological processes on the microbial assembly. Ecological processes of the (**a**) whole community, (**b**) core taxa, and (**c**) non-core taxa.

Ecological process partitioning revealed distinct differences in community assembly among the whole, core, and non-core microbial groups (Fig. 4a through c). In the whole community, homogeneous selection accounted for the largest proportion (57.89%), followed by undominated processes (29.55%) and dispersal limitation (11.61%), while heterogeneous selection (0.67%) and homogenizing dispersal (0.27%) contributed minimally (Fig. 4a). For the core taxa, all pairwise comparisons were classified as dispersal limitation under the βNTI-RC_bray_ framework (100%; Fig. 4b). In contrast, the non-core taxa were primarily associated with homogeneous selection (46.96%) and dispersal limitation (45.21%), with a smaller contribution from undominated processes (7.56%), whereas heterogeneous selection and homogenizing dispersal each accounted for only 0.13% (Fig. 4c). Overall, homogeneous selection represented the largest fraction of the assembly processes in the whole community. These classifications differed markedly between core and non-core taxa, with core communities primarily associated with dispersal limitation, whereas non-core communities reflect a combined influence of homogeneous selection and dispersal limitation.

Random forest analysis was performed to identify environmental variables associated with variation in microbial community assembly processes (Fig. S3; Table S8). Among these, NO_2_^−^-N had the highest variable importance score, followed by DO and T, whereas pH and ORP also contributed substantially. In contrast, PO_4_^3−^-P, SAL, TP, and TN showed moderate importance, while NH_4_^+^-N and NO_3_^−^-N were of relatively lower importance. Spatial analysis showed that |RC| was positively correlated with geographic distance (*R*^2^ = 0.045, *P* < 0.001; Fig. S4), indicating an increasing influence of dispersal limitation with spatial distance. Spearman correlation coefficient further showed a significant association between geographic distance and |RC| (*r* = 0.2827, *P* = 0.001; Table S9), supporting the contribution of spatial processes. Taken together, these analyses support the importance of both environmental and spatial factors in shaping microbial community composition and assembly patterns in the surface waters of the Xisha Islands. Notably, the relative distribution of ecological-process classifications differed between core and non-core taxa.

### Environmental associations with predicted microbial functional structure

**Fig 5:**
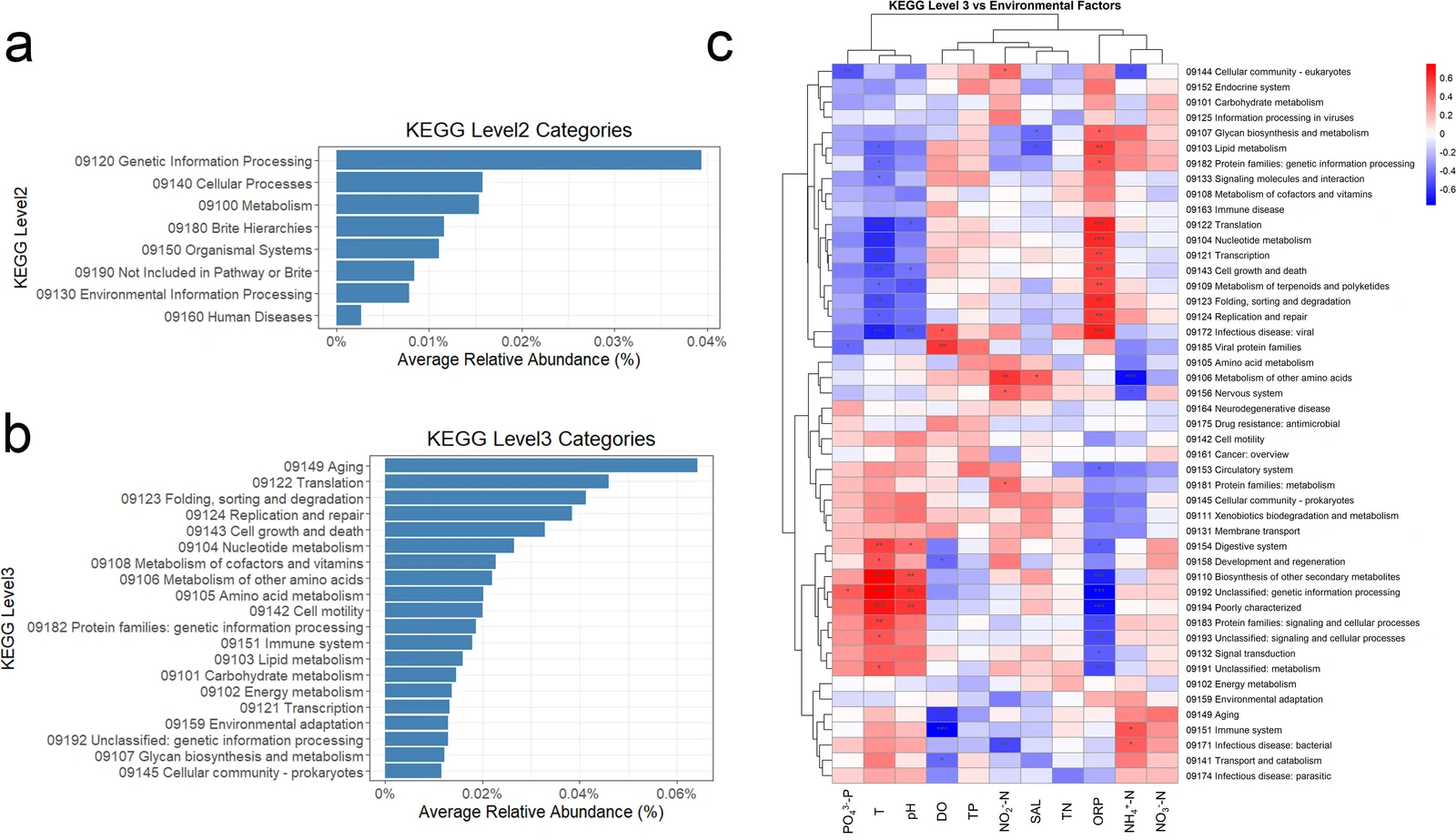
Relative abundances of microbial community functional genes and their relationships with environmental factors. Relative abundances of genes in (**a**) level 2 categories and (**b**) level 3 categories. (**c**) Correlations between the relative abundances of genes and environmental parameters. The color gradient on the right represents Spearman’s rank correlation coefficients, with more positive values (dark red) indicating stronger positive correlations and more negative values (dark blue) indicating stronger negative correlations. Asterisks denote significance levels: ***P < 0.001, **P < 0.01, and *P < 0.05.

KEGG functional annotation revealed distinct patterns in the functional composition of the microbial community across hierarchical levels (Fig. 5a and b). The mean NSTI was 0.181 ± 0.036. At KEGG level 2, genes associated with genetic information processing showed the highest relative abundance, followed by cellular processes and metabolism, whereas categories such as environmental information processing and human diseases accounted for relatively lower proportions (Fig. 5a). At level 3, aging and translation were among the most abundant functional categories, followed by folding, sorting and degradation, replication and repair, and cell growth and death. In addition, functions related to nucleotide metabolism, metabolism of cofactors and vitamins, and amino acid metabolism also exhibited relatively high abundances, suggesting considerable potential for fundamental metabolic and cellular maintenance functions (Fig. 5b).

Spearman correlation analysis revealed differential associations between environmental variables and microbial functional profiles (Fig. 5c). Among these variables, T, pH, and ORP were significantly correlated with a large proportion of functional categories (*P* < 0.05). In particular, functions related to genetic information processing (e.g., translation, transcription, and replication and repair) as well as metabolic pathways showed associations along gradients of T and ORP, while pH was also associated with multiple functional categories. In contrast, nitrogen-related variables (NO_2_^−^-N, NO_3_^−^-N, and NH_4_^+^-N) were associated with a smaller subset of functions, generally with lower consistency. DO, TN, phosphorus variables (PO_4_^3−^-P and TP), and SAL were associated with only a limited number of functional categories, suggesting comparatively weaker overall relationships. Overall, the functional structure of the microbial community was primarily associated with T, pH, and ORP, whereas other physicochemical variables showed more limited and function-specific associations, suggesting that a limited number of environmental variables may consistently influence the functional organization of microbial communities.

## DISCUSSION

Environmental and spatial factors are widely recognized as important drivers of marine microbial community organization, yet their respective roles remain incompletely understood, particularly in environments characterized by relatively low environmental heterogeneity (52, 53). Using the surface waters of the Xisha Islands as a natural system characterized by relatively narrow physicochemical gradients, we examined bacterial community composition, assembly patterns, and predicted functional structure. Despite the relatively narrow physicochemical variation among sites, both environmental and spatial factors were associated with microbial community composition, assembly patterns, and predicted functional structure. Notably, these associations differed between core and non-core taxa, which exhibited contrasting assembly patterns. In addition, a partial decoupling between taxonomic composition and functional potential was observed. Together, these findings support the importance of both environmental and spatial processes in structuring microbial communities and underscore the value of distinguishing core and non-core taxa when examining community composition, assembly, and potential ecosystem functioning in marine environments.

### Environmental filtering under low environmental heterogeneity

Our results indicate that physicochemical parameters varied within relatively narrow ranges across the study area, reflecting relatively low environmental heterogeneity (Fig. 1; Tables S1 and S2). Compared with estuarine, aquaculture, and coastal upwelling systems, where physicochemical variables often exhibit substantially broader physicochemical gradients (7, 54–56), the environmental variation observed across the Xisha Islands was comparatively narrow. Despite this, several environmental variables—particularly T, pH, and ORP—were consistently associated with microbial diversity (Fig. S2a), community composition (Fig. S2b through d, Fig. 3), assembly patterns (Fig. S3), and functional structure (Fig. 5c). Consistent with observations from the tropical North Pacific, even subtle environmental gradients can exert measurable influences on microbial communities in marine systems (57).

T emerged as the most influential variable across multiple analyses, including random forest and Mantel tests (Fig. 3a through c; Tables S5 through S7), highlighting its central role in microbial community composition variation (58–60). In tropical marine environments such as the Xisha Islands, where baseline temperatures are relatively high, even small variations may influence microbial metabolic dynamics and competitive interactions, potentially contributing to reduced diversity under elevated temperatures (5, 57).

SAL and pH were associated with microbial composition variation despite their limited range (Tables S1 and S2; Fig. 3a through c), suggesting that minor fluctuations in seawater acidity may contribute to niche differentiation, potentially through effects on enzyme activity, nutrient availability, and cellular homeostasis (61, 62). ORP also showed strong associations with both taxonomic and functional profiles (Tables S1 and S2; Fig. 3a through c and Fig. 5c), underscoring the relevance of redox conditions in shaping microbial metabolic potential (63–65). Variations in redox conditions can influence the availability of electron donors and acceptors, thereby affecting energy metabolism and biogeochemical processes (66).

Nitrogen-related variables, particularly NO_2_^−^-N, were also associated with community composition variation, as indicated by both random forest and correlation analyses (Fig. 3a through c; Fig. S2). As an intermediate in nitrification and denitrification, NO_2_^−^-N may reflect the intensity of nitrogen transformation processes and associated microbial activity (67).

Notably, these environmental associations were observed across the community as a whole, but their relative influence may differ among microbial subgroups, providing a basis for the contrasting ecological strategies observed between core and non-core taxa. Given the relatively low environmental heterogeneity of the study area, the measured physicochemical variables may also reflect broader oceanographic processes or covary with unmeasured environmental factors. Therefore, their associations with microbial community composition should be interpreted cautiously, as they do not necessarily indicate direct causal environmental selection.

### Joint roles of environmental and spatial processes

Beyond environmental influences, our results also suggest an important contribution of spatial processes to microbial community variation. Significant distance-decay relationships were observed across the whole, core, and non-core communities (Fig. 3d through f), indicating that community similarity decreased with increasing geographic distance, consistent with patterns reported in other marine, riverine, and lacustrine systems (57, 68, 69). Mantel tests further identified significant associations between geographic variables and community composition (Tables S5 through S7). In line with this, RC analysis showed a significant positive relationship between spatial distance and community turnover (Fig. S4), indicating an increasing influence of dispersal limitation with distance. Together, these results support a role for spatial processes in shaping microbial communities in the Xisha Islands, even within a relatively constrained geographic region (70, 71).

Ecological process partitioning further indicated that homogeneous selection accounted for the largest proportion of assembly processes in the whole community (Fig. 4a), suggesting that relatively similar environmental conditions across sites impose consistent selective pressures (11). This pattern is consistent with the observed low environmental heterogeneity and supports the importance of environmental filtering in structuring microbial communities in such systems (72). Taken together, these results suggest that microbial community variation reflects the combined influence of environmental and spatial processes.

### Divergent assembly patterns of core and non-core taxa

Compared with previous studies in tropical marine environments, the dominant microbial groups observed in the Xisha Islands were broadly consistent with those commonly reported from tropical oceans, suggesting that the microbial community composition in this region shares general characteristics with other tropical marine ecosystems (Fig. 2b) (73). Core taxa, defined by their high occurrence frequency and relatively stable abundance across samples (Fig. 2c and d), accounted for a small fraction of total richness but contributed disproportionately to community abundance (74, 75). In contrast, non-core taxa comprised the majority of richness and were more prominent in the microbial interaction network (Fig. 2g), consistent with observations in other ecosystems such as constructed wetlands (76). Core taxa likely represent relatively stable components of the community, whereas non-core taxa contribute disproportionately to community richness, network complexity, and potentially ecological interactions (77). Notably, the non-core community included *Bdellovibrionota*, a low-abundance but widely distributed lineage of predatory bacteria in marine ecosystems (78). Previous studies have shown that *Bdellovibrionota* is sensitive to environmental variation and may promote microbial diversity by regulating prey populations (79, 80). The occurrence of such taxa within the non-core community suggests that at least a subset of non-core microorganisms may respond more readily to localized microenvironmental variation while contributing disproportionately to community diversity and ecological interactions.

Building on the differential contributions of environmental and spatial processes, a key finding of this study is the contrasting assembly patterns between core and non-core taxa. Ecological process analysis revealed that core taxa were primarily associated with dispersal limitation, whereas non-core taxa were jointly influenced by homogeneous selection and dispersal limitation (Fig. 4b and c), indicating contrasting assembly patterns between these groups. Although core taxa occurred across all sampling sites, their relative abundances still varied spatially. Thus, the inferred association with dispersal limitation may primarily reflect spatially structured variation in relative abundance rather than differences in taxon occurrence. In contrast, non-core taxa exhibited greater sensitivity to both environmental and spatial factors. Their stronger distance-decay relationships, together with the relatively greater spatial component revealed by variation partitioning analysis, are consistent with the higher inferred contribution of dispersal limitation, suggesting stronger spatial structuring across sites (Fig. 3d through i, Fig. 4c) (81). The substantial contribution of homogeneous selection further suggests that similar environmental conditions may contribute to shaping non-core community turnover across sites. These findings support the view that occurrence-based group differentiation can reveal important ecological variation that may be obscured when microbial communities are analyzed as a whole (82).

### Decoupling between taxonomic composition and functional potential

Predicted functional profiles indicated that microbial communities were dominated by genes involved in genetic information processing and core metabolic pathways (Fig. 5a and b), indicating a strong emphasis on essential cellular functions. This pattern is commonly observed in oligotrophic marine environments, where efficient resource utilization and maintenance of basic cellular processes are critical for survival (58, 83).

Environmental variables, such as T, pH, and ORP, were associated with a wide range of functional categories (Fig. 5c), suggesting that environmental filtering operates not only at the taxonomic level but also at the functional level. However, nitrogen-related variables, despite their importance in shaping community composition, showed weaker and less consistent associations with functional structure. This mismatch between taxonomic and functional responses suggests a partial decoupling between community composition and functional potential. Such decoupling indicates a degree of functional redundancy within the microbial community, whereby different taxa may perform similar ecological functions (84, 85). As a result, changes in taxonomic composition do not necessarily translate into proportional shifts in functional potential. This functional redundancy may contribute to the maintenance of microbial functional stability despite taxonomic turnover and may enhance the resilience of ecosystem functioning under environmental variation, although this inference requires direct functional validation (86, 87).

### Conclusion

Microbial community composition, assembly patterns, and predicted functional structure in the surface waters of the Xisha Islands were associated with both environmental and spatial factors despite relatively low environmental heterogeneity. However, these associations varied between the core and non-core communities. Notably, assembly analyses revealed contrasting patterns: core-community comparisons were classified as dispersal limitation, whereas non-core comparisons were primarily associated with homogeneous selection and dispersal limitation. Furthermore, the partial decoupling between taxonomic composition and predicted functional potential suggests that functional stability may be maintained despite taxonomic turnover. Together, these findings highlight the ecological importance of distinguishing core and non-core taxa for understanding microbial community assembly in tropical marine surface waters.

## Supplementary Material

Reviewer comments

## Data Availability

All sequencing data used in this study are available in the NCBI Short Read Archive (https://www.ncbi.nlm.nih.gov/sra) under Bioproject PRJNA1460329.

## SUPPLEMENTAL MATERIAL

Fig. S1 spectrum.02178-26-s0001.tif Composition of microbial communities in the surface waters of the Xisha Islands.

Fig. S2 spectrum.02178-26-s0002.tif Environmental and geographic correlates of microbial alpha diversity and composition.

Fig. S3 spectrum.02178-26-s0003.tif Environmental drivers of microbial community assembly.

Fig. S4 spectrum.02178-26-s0004.tif Geographic drivers of microbial community assembly.

Supplemental legends spectrum.02178-26-s0005.docx Legends for Fig. S1 to S4 and Tables S1 to S9.

Supplemental tables spectrum.02178-26-s0006.xlsx Tables S1 to S9.

OPEN PEER REVIEW reviewer-comments.pdf An accounting of the reviewer comments and feedback.

ASM does not own the copyrights to Supplemental Material that may be linked to, or accessed through, an article. The authors have granted ASM a non-exclusive, world-wide license to publish the Supplemental Material files. Please contact the corresponding author directly for reuse.
