# Supplementary material for: Divergent assembly patterns of core and non-core microbial taxa under low environmental heterogeneity in the surface waters of the Xisha Islands
Source: Microbiol Spectr. 2026 Aug 14;14(9):e02178-26. doi: 10.1128/spectrum.02178-26 (PMC13532294; doi:10.1128/spectrum.02178-26)
Supplement: Supplementary file 1 [file reviewer-comments.pdf]

ASM | Microbiology Spectrum

Divergent assembly patterns of core and non-core microbial taxa under low environmental heterogeneity in the surface waters of the Xisha Islands

Zhixuan Deng, Renjun Zhou, Dongwei Hou, Shenzheng Zeng, Shaoping Weng, Jianguo He, and Zhijian Huang

Corresponding Author(s): Zhixuan Deng, Sun Yat-sen University - Zhuhai Campus

Review Timeline:

Submission Date:	July 10, 2026
Editorial Decision:	July 20, 2026
Revision Received:	July 21, 2026
Accepted:	July 24, 2026

Editor: Jan Claesen

Reviewer(s): The reviewers have opted to remain anonymous.

Transaction Report:

(Note: With the exception of the correction of typographical or spelling errors that could be a source of ambiguity, letters and reports are not edited. The original formatting of letters and referee reports may not be reflected in this compilation.)

DOI: https://doi.org/10.1128/spectrum.02178-26

Re: Spectrum02178-26 (**Divergent assembly patterns of core and non-core microbial taxa under low environmental heterogeneity in the surface waters of the Xisha Islands**)

Dear Mr. Zhixuan Deng:

Thank you for the privilege of reviewing your work. I would like to thank you for carefully addressing the Reviewers' comments from your prior submission to AEM, substantially improving the accessibility and rigor of your paper. I am pleased to inform you that your manuscript has been editorially accepted for publication. However, there are a few additional questions in the submission form that need to be answered before your manuscript is officially accepted. Once these are completed, please return your submission so that I can move your paper forward to acceptance.

Please return the manuscript within 60 days; if you cannot complete the submission questions within this time period, please contact me.

Thank you for submitting your paper to Spectrum.

Sincerely,
Jan Claesen
Editor
Microbiology Spectrum

Reviewer #1 (Comments for the Author):

This manuscript investigates microbial community composition, core/non-core taxa, assembly processes, and predicted functional potential in surface waters around the Xisha Islands. The study addresses an interesting and timely question in marine microbial ecology: how microbial communities assemble under relatively low environmental heterogeneity, and whether core and non-core taxa follow different ecological mechanisms. The dataset has value, especially because tropical island-associated surface waters remain less studied than coastal, estuarine, or open-ocean systems. The distinction between core and non-core taxa is also potentially useful. However, several concerns require attention before publication.

Response: We sincerely thank the reviewer for the positive evaluation of our study. We appreciate the recognition of the value of the dataset, the ecological relevance of the Xisha Islands surface-water ecosystem, and the potential usefulness of distinguishing between core and non-core taxa. We have carefully considered all comments and suggestions and have revised the manuscript accordingly. Detailed responses to each point are provided below.

1. The manuscript states that a total of 5,005 ASVs were detected, but later reports 42 core ASVs and 3,605 non-core ASVs. These numbers do not add up. If filtering was applied before defining core/non-core taxa, this must be explicitly described.

Response: We thank the reviewer for identifying this inconsistency and apologize for the confusion caused. The reported value of 5,005 ASVs was a typographical error introduced during manuscript preparation. The correct total number of ASVs included in the analyses was 3,647, comprising 42 core ASVs and 3,605 non-core ASVs. No additional filtering step was performed prior to the definition of core and non-core taxa.

Changes made: Results (Lines 241).

2. The manuscript reports that core taxa were assembled by 100% dispersal limitation. This is a striking result, but it requires much more cautious interpretation. Core taxa, by definition, occurred in all samples and were relatively abundant. Therefore, interpreting their turnover as dispersal limitation is not straightforward. it needs deeper

discussion. Maybe, you can try the newest method of null model analysis of iCAMP(https://github.com/DaliangNing/icamp1).

Response： We thank the reviewer for this insightful comment. We agree that the interpretation of dispersal limitation in core taxa requires careful consideration because these taxa were detected in all samples and represented a substantial proportion of community abundance. In the βNTI-RCbray framework, dispersal limitation reflects spatially structured compositional turnover rather than the complete absence of dispersal. Therefore, we have revised the manuscript to avoid overly deterministic interpretations and have expanded the Discussion to clarify the ecological meaning and potential limitations of this result. Specifically, we now emphasize that the observed pattern may reflect spatially constrained abundance variation among widely distributed taxa rather than restricted occurrence per se. We also acknowledge that the relatively small number of core ASVs (42) may influence the robustness of this result and that future studies applying complementary approaches, such as iCAMP, would be valuable for further validation. However, because iCAMP was not included in the original analytical framework and would require substantial reanalysis beyond the scope of the present revision, we did not incorporate it in the current study.

Changes made: Discussion (Lines 474–477).

3. The manuscript repeatedly emphasizes "low environmental heterogeneity." This is plausible, but it should be demonstrated more rigorously. For example, temperature varied from 29.5 to 31.5{degree sign}C, which is a 2{degree sign}C range. In tropical marine systems, a 2{degree sign}C difference can be ecologically meaningful. Similarly, pH ranged from 7.85 to 8.22, which is not negligible. The manuscript can still argue that heterogeneity is relatively low compared with estuaries, aquaculture ponds, or coastal pollution gradients, but the claim should be more explicitly justified. I suggest add the coefficients of variation for environmental variables, PCA or clustering of environmental variables, and comparison with broader coastal/open-ocean gradients from the literature.

Response: We thank the reviewer for this constructive suggestion. We agree that the characterization of "low environmental heterogeneity" should be more explicitly justified. In response, we have strengthened the manuscript in two ways. First, we

expanded both the Introduction and the Discussion by incorporating comparisons with marine environments characterized by substantially greater environmental heterogeneity (e.g., estuaries, coastal upwelling systems, and broader oceanic gradients), thereby clarifying that "low environmental heterogeneity" is intended as a relative rather than an absolute description. Second, we added a new Supplementary Table S2 summarizing the mean±SD, range, and coefficient of variation (CV) for all measured physicochemical variables, providing a quantitative assessment of environmental variability across the sampling sites. We believe these revisions provide a more rigorous justification for the use of the term "low environmental heterogeneity."

Changes made: Introduction (Lines 81–90); Discussion (Lines 389–392); Supplementary Table S2.

4. Mantel tests show associations between community dissimilarity and environmental/geographic distance, but they do not partition variance or establish relative contributions of environment and space. The manuscript uses language such as "relative contributions" and "environmental and spatial processes jointly structure microbial communities," but the analyses do not fully quantify this. The author should add the variation partitioning using environmental variables and spatial eigenvectors or some more strong method.

Response: We thank the reviewer for this constructive suggestion. We agree that Mantel tests alone do not quantify the independent contributions of environmental and spatial factors. Following the reviewer's recommendation, we performed variation partitioning analysis (VPA) to quantify the unique and shared fractions of community variation explained by environmental and geographic variables for the whole, core, and non-core communities. The VPA results showed that environmental variables explained a substantially larger proportion of community variation than geographic variables across all community types, while the non-core community exhibited a relatively greater spatial component than the core community. We have incorporated these analyses into the revised manuscript and revised the corresponding text accordingly.

Changes made: Figure 3g–I; Materials and Methods (Lines 206–210); Results (Lines 303–307); Discussion (Lines 478–482) and Abstract (Lines 35–39).

5. NSTI values are mentioned but never reported - this is critical for assessing prediction reliability.

Response: We thank the reviewer for this important comment. We agree that reporting NSTI values is important for evaluating the reliability of PICRUSt2-based functional predictions. In the revised manuscript, we have added the abundance-weighted NSTI values across all samples. The mean NSTI was 0.181±0.036, providing an assessment of the confidence of the predicted functional profiles.

Changes made: Results (Lines 344–345).

6. Line 19: Check email spelling: "mial.sysu.edu.cn" appears to be a typo.

Response: We thank the reviewer for pointing out this typographical error. The email address has been corrected in the revised manuscript.

Changes made: The corresponding author's email address has been corrected.

7. Line 134: 2.0 liter should be 2.0 liters.

Response: We thank the reviewer for this careful observation. The grammatical error has been corrected in the revised manuscript.

Changes made: "2.0 liter" has been corrected to "2.0 liters" (Line 144).

8. Line 139: dissolve oxygen should be dissolved oxygen.

Response: We thank the reviewer for this careful correction. The term has been revised in the manuscript.

Changes made: “dissolve oxygen” has been corrected to “dissolved oxygen” (Line 150).

9. Line 156: Novegene shoule be Novogene

Response: We thank the reviewer for pointing out this typographical error. The company name has been corrected in the revised manuscript.

Changes made: “Novegene” has been corrected to “Novogene” (Line 170).

10. Line 201: "metagenomic functional prediction consequences" is awkward. Use "predicted functional profiles."

Response: We thank the reviewer for this helpful suggestion. The phrase has been revised to improve clarity and readability.

Changes made: “metagenomic functional prediction consequences” has been replaced with “predicted functional profiles” (Line 220).

11. Line 232: unclassfied should be unclassified

Response: We thank the reviewer for pointing out this typographical error. The spelling has been corrected in the revised manuscript.

Changes made: “unclassfied” has been corrected to “unclassified” (Line 246).

12. Line 808: "geographic drivers" should be capitalized consistently in figure titles.

Response: We thank the reviewer for this careful suggestion. We have standardized the capitalization to ensure consistency across all figure titles.

Changes made: “geographic drivers” has been corrected to “Geographic drivers” in the relevant figure title (Line 867).

Reviewer #2 (Comments for the Author):

This study provides a valuable exploration of microbial biogeography in the Xisha Islands, a unique and ecologically significant tropical oceanic island system in the South China Sea. The research is well-conceived, filling a critical knowledge gap as previous studies in this region have primarily focused on seabird communities or sediment-associated microbes rather than surface-water bacterioplankton. The author's approach of distinguishing between "core" and "non-core" taxa to reveal divergent assembly mechanisms under low environmental heterogeneity is both timely and scientifically significant. This framework offers quantitative insights into how stable marine environments maintain microbial diversity and functional potential. Although this study is sound, some further improvements are still needed before it can be accepted.

Response: Thank you for the positive evaluation and constructive comments. We sincerely appreciate the reviewer's recognition of the novelty and significance of our study, particularly the value of investigating surface-water bacterioplankton in the Xisha Islands and the ecological framework comparing core and non-core taxa under relatively low environmental heterogeneity. We are also grateful for the thoughtful suggestions provided throughout the review. We have carefully considered each comment and have revised the manuscript accordingly to improve its clarity, methodological rigor, and overall presentation. We believe these revisions have substantially strengthened the manuscript and hope that the revised version satisfactorily addresses all of the reviewer's concerns.

1. The current spatial distribution of the 13 sampling sites appears completely random, failing to demonstrate fine-scale sampling. If the authors aimed to resolve "finer-scale" ecological processes, it is suggested that the authors provide a more explicit rationale for the site selection, explaining how this specific layout captures the intended fine-scale spatial variations or defining the exact spatial boundaries the study intends to represent.

Response: We thank the reviewer for this important comment. We would like to clarify that the sampling sites were not randomly selected. The sampling design was established to maximize spatial coverage of the surface waters surrounding the Xisha Islands, with sites distributed across the central and peripheral areas of the study region.

Due to operational constraints associated with sampling from a research vessel, some areas were inaccessible, and the final sampling locations were determined based on navigational accessibility and safety considerations.

We also appreciate the reviewer's concern regarding the spatial scale of the study. The term "finer-scale" in the original manuscript was intended to describe the regional spatial extent of the Xisha Islands relative to studies conducted across systems with more pronounced environmental gradients or much broader spatial scales (e.g., estuarine ecosystems, coastal upwelling regions, or global ocean surveys). We have revised the description of the sampling design to better clarify the spatial extent of our study. Specifically, we now state that the sampling sites were distributed across the Xisha Islands to represent the regional spatial extent of the surface-water system and capture naturally occurring environmental variation among sampling locations.

Changes made: Material and methods (Lines 140–144).

2. While the authors demonstrate that physicochemical parameters (e.g., temperature and salinity) vary within narrow ranges, a constructive addition would be to quantitatively compare these ranges with high-heterogeneity systems (such as estuaries or upwelling zones). This would strengthen the definition of "low heterogeneity" and provide a clearer context for why the observed subtle environmental gradients are ecologically meaningful.

Response: We thank the reviewer for this constructive suggestion. We agree that the characterization of "low environmental heterogeneity" should be more explicitly justified. In response, we have strengthened the manuscript. We expanded both the Introduction and the Discussion by incorporating comparisons with marine environments characterized by substantially greater environmental heterogeneity (e.g., estuaries, coastal upwelling systems, and broader oceanic gradients), thereby clarifying that "low environmental heterogeneity" is intended as a relative rather than an absolute description. We believe these revisions provide a more rigorous justification for the use of the term "low environmental heterogeneity."

Changes made: Introduction (Lines 81–90); Discussion (Lines 387–392).

3. The study currently utilizes SILVA version 123 for taxonomic classification. Given that the differentiation between core and non-core taxa-particularly the highly diverse non-core group-relies heavily on taxonomic accuracy, it is recommended to update the analysis using a more recent version of the SILVA database (e.g., v138.1 or newer) to ensure the findings reflect the most current microbial systematic standards.

Response: We thank the reviewer for this careful observation. After verifying our analysis pipeline, we confirmed that taxonomic classification was performed using the SILVA database version 138, rather than version 123 as incorrectly stated in the manuscript. This was a typographical error in the Methods section only and does not affect any of the analyses or conclusions presented in this study. The manuscript has been corrected accordingly.

Changes made: The SILVA database version has been corrected from **v123** to **v138** in the Methods section (Line 178).

4. The finding that core taxa (consisting of 42 ASVs) are governed exclusively by dispersal limitation is intriguing. The authors should provide a more detailed interpretation of what "dispersal limitation" implies for taxa already detected in all samples-for instance, whether it refers to the stochasticity of abundance fluctuations. Additionally, considering the small number of core ASVs, performing a sensitivity or robustness analysis on the null model results would add significant weight to these conclusions.

Response: We thank the reviewer for this insightful comment. We agree that the ecological interpretation of dispersal limitation for widely distributed core taxa requires clarification. In the revised Discussion, we have clarified that although the core taxa were detected across all sampling sites, their relative abundances still varied spatially. Therefore, the inferred association with dispersal limitation should be interpreted as reflecting spatially structured variation in relative abundance rather than differences in taxon occurrence.

Regarding the reviewer's suggestion of a sensitivity or robustness analysis, we acknowledge that the core community comprised only 42 ASVs. This relatively small number reflects the stringent and predefined criterion used to define the core microbiota,

which was applied consistently throughout the study. While sensitivity analyses based on alternative core-taxon definitions could provide additional information, they would require redefining the core and non-core communities and repeating all downstream analyses, which is beyond the scope of the present study.

Changes made: Discussion (Lines 474–477).

5. The authors noted a partial decoupling between taxonomic composition and functional potential. Expanding the discussion on how functional redundancy specifically buffers the Xisha Islands' ecosystem against environmental fluctuations would be highly beneficial for understanding community stability.

Response: We thank the reviewer for this valuable suggestion. We agree that functional redundancy is an important perspective for understanding taxonomic–functional decoupling. In the revised Discussion, we have retained and refined our discussion of functional redundancy, highlighting its potential role in maintaining relatively stable predicted functional potential despite taxonomic turnover.

However, the primary focus of this study is to elucidate how environmental and spatial factors shape microbial community composition and assembly patterns, particularly the contrasting responses of core and non-core taxa. Since functional profiles were inferred based on PICRUSt2, further exploration of specific functional mechanisms and their contributions to ecosystem stability would require direct evidence from metagenomic, metatranscriptomic, or other functional approaches.

6. The non-core taxa contribute disproportionately to the complexity of the co-occurrence network. The authors could further discuss whether these taxa represent "ecological specialists" that respond to micro-niche variations, contrasting with the more stable "generalist" core taxa.

Response: We thank the reviewer for this insightful suggestion. We agree that considering the ecological characteristics of core and non-core taxa provides additional context for interpreting their contrasting assembly patterns. In the revised Discussion, we have expanded the interpretation of the non-core community by discussing that certain non-core lineages, such as the predatory phylum *Bdellovibrionota*, are sensitive

to environmental variation and may respond more readily to localized micro-niche conditions. These characteristics may partly explain the greater network complexity and stronger spatial structuring observed for the non-core community. However, we have avoided directly classifying core and non-core taxa as ecological generalists or specialists, as such classifications would require dedicated niche-based analyses beyond the scope of the present study.

Changes made: Discussion (Lines 461–468).

Reviewer #3 (Comments for the Author):

1. Characterizing microbial community composition in specific habitats is also an important scientific question. It would be valuable to discuss whether the microbial community composition in this region differs from that reported in other tropical marine environments.

Response: We thank the reviewer for this valuable suggestion. We agree that placing the microbial community composition of the Xisha Islands in the context of other tropical marine environments helps improve the ecological interpretation of our findings. Accordingly, we have expanded the Discussion by comparing our results with previous studies from tropical oceans. Overall, the dominant microbial groups identified in the Xisha Islands were broadly consistent with those commonly reported in tropical marine environments, suggesting that the microbial community composition in this region shares general characteristics of tropical marine ecosystems and is representative of this habitat type.

Changes made: Discussion (Lines 449–453).

2. Is the predatory phylum Bdellovibrionota commonly found in marine environments? Please further discuss its potential ecological significance.

Response: We thank the reviewer for this valuable suggestion. We agree that the ecological significance of *Bdellovibrionota* deserves further discussion. Accordingly, we have expanded the Discussion to clarify that *Bdellovibrionota* is a low-abundance but widely distributed lineage of predatory bacteria in marine ecosystems. We further discuss its reported ecological roles in regulating prey populations and maintaining microbial diversity, and relate these characteristics to its occurrence within the non-core community in the Xisha Islands.

Changes made: Discussion (Lines 461–468).

3. The study relies on PICRUSt2-based functional prediction rather than metagenomic sequencing. The conclusions regarding microbial functional structure may therefore be indirect and potentially uncertain. Please clarify this limitation and avoid overinterpretation of functional results.

Response: We thank the reviewer for this important comment. We agree that PICRUSt2-based functional prediction provides insights into the predicted functional potential of microbial communities but does not directly measure microbial functional activity. To address this concern, we have revised the manuscript to avoid overinterpretation of functional results. Specifically, we have consistently added terms such as "predicted" and "potential" when describing microbial functional profiles throughout the manuscript. In addition, we have added a discussion acknowledging the limitation of PICRUSt2-based functional inference and emphasizing that the ecological implications of predicted functional patterns require further validation using direct functional approaches.

Changes made: Discussion (Lines 503–506).

4. In the Introduction section, it is recommended to include additional references discussing the differential effects of environmental and geographic factors on microbial community assembly to better support the statement: "However, the relative importance of environmental and spatial processes in shaping marine microbial communities remains debated."

Response: We thank the reviewer for this helpful comment. We agree that the statement regarding inconsistent patterns across ecosystems should be explicitly supported by the relevant literature. To address this, we have revised the text to clarify that the inconsistency refers to the contrasting relative contributions of deterministic and stochastic processes to the assembly of core and non-core taxa reported across different ecosystems, and we have cited the relevant references immediately following this statement. We believe this revision more clearly conveys the intended meaning and strengthens the support for our statement.

Changes made: Introduction (Lines 99–104).

5. Please ensure the correct formatting of symbols and punctuation throughout the manuscript, such as in "Bray-Curtis similarity" and "Distance-decay".

Response: We thank the reviewer for this helpful comment. We have carefully checked the entire manuscript and standardized the formatting of symbols, punctuation, and

compound terms to ensure consistency throughout. We believe these revisions improve the clarity and consistency of the manuscript.

Changes made: Manuscript (throughout).

6. Lines 209-210: The geographic coordinates are provided in Supplementary Table 1 rather than Supplementary Table 3.

Response: We thank the reviewer for pointing out this error. We have corrected the manuscript and ensured that the geographic coordinates are now correctly referred to in Supplementary Table 1 instead of Supplementary Table 3. We have also carefully checked the manuscript to ensure that no similar referencing errors remain.

Changes made: Materials and Methods (Lines 228–229).

7. Lines 211-224: It is recommended that all physicochemical parameters be presented in the format of mean {plus minus} standard deviation to improve the conciseness of the Results section.

Response: We thank the reviewer for this helpful suggestion. We have revised the manuscript accordingly and standardized the presentation of all physicochemical and nutrient parameters in the Results section to mean±standard deviation. This revision improves the clarity, conciseness, and consistency of the Results.

Changes made: Results (Lines 228–237).

8. The font size in some figure legends is too small. Please increase the font size to improve readability.

Response: We thank the reviewer for this helpful suggestion. We have increased the font size in all figure legends to improve readability.

Changes made: The font sizes in all figure legends have been increased in the revised figures.

9. The term "alpha diversity" is inconsistently formatted throughout the manuscript, with some instances capitalized and others not. Please ensure consistent formatting

throughout the text.

Response: We thank the reviewer for pointing out this inconsistency. We have carefully checked the manuscript and standardized the formatting of the term "alpha diversity" throughout the text to ensure consistency.

Changes made: The formatting of "alpha diversity" has been standardized throughout the manuscript.

10. Line 266: "significantly correlated" would be more appropriate than "significantly associated". Please check and revise similar expressions throughout the manuscript.

Response: We thank the reviewer for this helpful suggestion. We have carefully checked the manuscript and revised the expressions related to statistical relationships to ensure that "significantly correlated" is used where appropriate.

11. Line 354: Please avoid using the term "clear," as it is overly subjective in this context.

Response: We thank the reviewer for this helpful suggestion. We agree that the term "clear" is subjective in this context. Accordingly, we have removed this term to provide a more objective description of the observed associations.

Changes made: The term "clear" has been removed from the corresponding sentence in the Results (Lines 359).

12. In Figure 1, the dashed boxes overlap with parts of the text. Please adjust the figure layout to improve readability.

Response: We thank the reviewer for pointing out this layout issue. We have adjusted the figure layout to eliminate the overlap between the dashed boxes and the text, thereby improving readability.

Changes made: The layout of Figure 1 has been revised to avoid overlap between the dashed boxes and the text.

13. It is recommended to add clearer labels or annotations to Figures 3 and 4 to facilitate

distinction between the results for the core and non-core taxa.

Response: We thank the reviewer for this helpful suggestion. We have revised Figures 3 and 4 by adding clearer labels to facilitate distinction between the results for the whole, core, and non-core communities and improve the overall readability of the figures.

Changes made: Figures 3 and 4.

14. It is recommended to use consistent abbreviations for physicochemical parameters throughout the manuscript.

Response: We thank the reviewer for this helpful suggestion. We have carefully checked the manuscript and standardized the abbreviations of all physicochemical parameters throughout the text to ensure consistency.

15. For the network in Figure 2, it is recommended to add more legends to facilitate interpretation of the meanings represented by different node and edge colors.

Response: We thank the reviewer for this helpful suggestion. We have revised Figure 2 by adding additional legend information to clearly explain the meanings of the different node and edge colors, thereby improving the interpretability of the network.

Changes made: Additional legends have been added to Figure 2 to clarify the meanings of the node and edge colors.

Reviewer #4 (Comments for the Author):

Comments for Deng et al. 2026

The manuscript titled "Divergent assembly mechanisms of ore and non-core microbial taxa under low environmental heterogeneity in the Xisha Islands" by Deng et al. presents a study of surface-water microbial communities sampled from the Xisha Islands with a focus on core and non-core taxa. Its aims to examine how environmental and spatial factors shape community structure, assembly, and functional potential. In my opinion, the results represent only a snapshot of ever-changing microbial communities that can be affect by some many analysed and non-analysed factors. Furthermore, the description of the habitat is really limited (I still don't know what the sampling sites are). The findings are heavily dependent on the snapshot observation and statistical analyses, which inherently are limited by what you measured. Some of the methods (e.g., functional prediction using PICRUSt2) are not solid. Studies of this type (amplicon + network + statistical analysis) failed to provide convincing evidence that the results can survive at different sampling size, variable measured, sampling time/season, and so on. Therefore, although the interpretation on this particular set of samples (with the particular measurements) from this particular sampling area may be solid, they cannot be applied to anything else.

Responses： We sincerely thank the reviewer for the thoughtful overall assessment of our manuscript. We agree that this study represents a survey of microbial communities collected from a specific region during a single sampling campaign, and that the observed patterns should therefore be interpreted within the context of the sampled system. Our objective was to investigate microbial community composition, assembly patterns, and predicted functional potential in the surface waters of the Xisha Islands, and to evaluate their associations with measured environmental and spatial factors. Such studies contribute empirical evidence from tropical marine environments that can be compared across ecosystems and integrated into broader frameworks of microbial community assembly and biogeography.

To address the reviewer's concerns, we have revised the manuscript to adopt more cautious language throughout. Specifically, we replaced several causal expressions with more conservative descriptions, moderated the interpretation of community assembly

patterns, and avoided extending our conclusions beyond the scope of the present dataset. We also expanded the description of the study area and sampling sites in the Introduction and Materials and Methods to better describe the environmental context of the sampling region. In addition, we explicitly clarify that the functional analyses are based on PICRUSt2 predictions and therefore represent predicted functional potential rather than direct metagenomic measurements.

We believe these revisions more accurately reflect the scope and limitations of the study while preserving the main findings supported by our data.

Major comments

I am not an ecologist and the authors really left me behind by using their jargons (probably they are only jargons to me). For example, what do the "Random Forest Variable Importance (%IncMSE)" in Fig. 3 mean? In the figure legend, the authors described them as "Random forest analysis showing the relative importance of environmental variables and geographical distance in explaining the Bray-Curtis dissimilarity of the (a) whole community, (b) core taxa, and (c) non-core taxa.". Relative importance in/to what? Why the sum of these variables is not 100%? I really have no idea how these results were generated and what they mean.

Responses: We appreciate the reviewer for pointing out that the description of the random forest analysis was not sufficiently clear for readers who are less familiar with ecological statistical methods. We agree that our original use of the term "relative importance" may have been ambiguous and could be confused with a percentage contribution. To improve clarity, we have revised the manuscript throughout by replacing this terminology with "variable importance scores (%IncMSE)" or equivalent descriptions (e.g., "the variable with the highest importance score" or "ranked among the most important variables"), which more accurately reflect the output of the random forest model.

In addition, we have revised the figure legends to explicitly state that the random forest results are presented as variable importance scores (%IncMSE) and clarified that higher %IncMSE values indicate a greater contribution of a variable to model prediction. We have also revised the corresponding Results section to describe the

rankings of variables more precisely and consistently, avoiding potentially misleading expressions such as "relative importance."

We hope these revisions improve the accessibility of the manuscript for readers who are less familiar with ecological statistical terminology.

Changes made: Materials and Methods (Lines 211–215), Results (Lines 285–292; 328–341), Figure 3 and Figure S3 legend.

What does "low environmental heterogeneity" mean? What are the criteria to determine if the heterogeneity of an environment is high or low? Why does it matter of us to understand the "low environmental heterogeneity"? In other words, what would we miss (or what could be wrong) if we do not understand it? We need answers to these questions to better understand this type of ecological science with heavy reliance on snapshot observations and statistical analyses.

Responses： We thank the reviewer for this insightful comment. We agree that the rationale for studying microbial community assembly under relatively low environmental heterogeneity should be more clearly explained. In the revised Introduction, we now clarify that "low environmental heterogeneity" is used in a relative sense by explicitly contrasting our study system with marine environments characterized by much stronger environmental gradients, including estuarine ecosystems, coastal upwelling regions, and global ocean surveys, where most previous studies have been conducted.

We also wish to clarify the ecological significance of this study. Most current knowledge of microbial community assembly has been developed from systems with pronounced environmental heterogeneity, where strong environmental gradients can dominate community variation and potentially obscure the influence of more subtle environmental and spatial processes. Studying a relatively stable marine system therefore allows us to evaluate whether environmental and spatial processes remain associated with microbial community assembly when large environmental gradients are absent. Such studies provide complementary evidence that helps refine and broaden ecological models of microbial community assembly across different environmental contexts, rather than replacing findings from more heterogeneous ecosystems.

Changes made: Introduction (Lines 81–90).

The nature of the sampling sites is unclear. For each sampling site, what is the distance to shore? How was the sampling done? By sanding on shore or on a boat/canoe? I am confused because I (and probably many others) don't know what Xishan Islands are. Are the samples mainly derived from open ocean or coastal seawater, or just beaches? How important is the ecosystem below affect the surface-water microbial communities?

Responses： We thank the reviewer for this helpful comment. We agree that the original description of the sampling sites was not sufficiently detailed, particularly for readers who may be unfamiliar with the Xisha Islands and the characteristics of the sampling locations. To improve clarity, we have expanded the Sample collection and physicochemical analysis section by providing additional information on the sampling design and sampling procedure.

Specifically, we now clarify that the sampling sites were distributed across the Xisha Islands to represent the regional spatial extent of the surface-water system and to capture naturally occurring environmental variation among locations. We have also specified that all surface-water samples were collected from a research vessel at the designated sampling sites.

Changes made: Materials and Methods, Sample collection and physicochemical analysis (Lines 140–145): Expanded the description of the sampling design and clarified the sampling procedure.

Supplementary Table S1: Geographic coordinates of all sampling sites are provided.
"quantitative insights into ..." (Line 47 in Abstract). Are you sure that the numbers in Abstract [e.g., "homogeneous selection (46.96%) and dispersal limitation (45.21%)"] can be generalized to other tropical marine surface waters? I doubt that these numbers will be different if only one more sample were analysed. In other words, I cannot see how these numbers can be consistent and robust across repeated analyses.

Responses： We thank the reviewer for this helpful comment. We agree that the numerical estimates reported in this study represent observations from the investigated system and should not be interpreted as universally applicable values for tropical

marine environments. Accordingly, we have revised the Abstract to remove the phrase "quantitative insights into" and instead state that the study " This study highlights the importance of considering microbial subcommunities when investigating community assembly in marine ecosystems " This revised wording better reflects that our conclusions are based on the observed patterns in the Xisha Islands while contributing to the broader understanding of microbial community assembly.

Changes made: Abstract (Lines 48–49).

Minor comments

In Abstract, the sample number and how they were sampled from where should be described. Also, the investigated ecosystem should be explicitly described in the Title and Abstract. I was really confused what the samples are until Line 108. Microbial (core and non-core under low environmental heterogeneity) taxa in the Xisha Islands are just too ambiguous. Are they from the surface water, coastal sediment, or even some terrestrial habitats? Obviously, samples from different habitats should be interpreted differently.

Response: We thank the reviewer for this valuable comment. We agree that the investigated ecosystem and sampling information should be presented more explicitly to help readers immediately understand the scope of the study. In response to this suggestion, we have revised both the Title and the Abstract. The Title now explicitly specifies that the study focuses on the surface waters of the Xisha Islands, thereby clearly identifying the investigated ecosystem. In the Abstract, we have added the total number of samples analyzed and clarified that the samples were collected from the surface waters of the Xisha Islands (South China Sea). In addition, we have further expanded the description of the sampling sites and sampling procedures in the Materials and Methods section by specifying that samples were collected from surface waters surrounding the Xisha Islands from a research vessel, providing the geographic coordinates of all sampling sites, and including additional information describing the study area. We believe these revisions make both the investigated ecosystem and the sampling strategy clear to readers from the beginning of the manuscript.

Changes made: Title; Abstract (Lines 27–31); Materials and Methods (Lines 140–145).

Revision made:

What does "spatial processes" refer to, and also "environment processes" (e.g., Lines 43-44)? I am not an expert on ecology and thus may not be well familiar with this term.

Response: We thank the reviewer for this helpful suggestion. We agree that the terms "environmental processes" and "spatial processes" may benefit from brief clarification to improve readability. To improve accessibility, we have added brief explanations when these terms are first introduced in the Introduction. Specifically, we clarify that environmental processes refer to ecological processes related to environmental conditions, whereas spatial processes refer to ecological processes related to spatial structure and geographic distance. We believe these additions improve the clarity of the terminology for a broader readership while maintaining the conciseness of the manuscript.

Changes made: Introduction (Lines 86–90).

How long were the samples stored at 4 °C before nutrient analysis? Does it affect the nutrient measurements? I think the standard protocol would be frozen at -20 °C (Becker, S., Aoyama, M., Woodward, E.M.S., Bakker, K., Coverly, S., Mahaffey, C., and Tanhua, T. (2020) GO-SHIP repeat hydrography nutrient manual: The precise and accurate determination of dissolved inorganic nutrients in seawater, using continuous flow analysis methods. Frontiers in Marine Science 7: 581790.).

Responses： We thank the reviewer for raising this important point regarding nutrient sample preservation.

To minimize potential changes in nutrient concentrations during storage, seawater samples for dissolved inorganic nutrient analyses (NH_4^+-N, NO_2^--N, NO_3^--N, and PO_4^{3-}-P) were first passed through 0.22 μm membrane filters to remove the vast majority of microbial cells, thereby greatly reducing biological transformation during storage. The filtered samples were then stored at 4 °C until laboratory analysis following the cruise. Total nitrogen (TN) and total phosphorus (TP) were determined from unfiltered samples following the analytical protocol of the instrument.

Importantly, all samples were collected during the same cruise, processed using the

same protocol, stored under identical conditions, and analyzed immediately after returning to the laboratory. Therefore, although we acknowledge that prolonged storage is not ideal, any potential effects would be expected to influence all samples consistently rather than introduce systematic differences among sampling sites.

To improve transparency, we have clarified the sample preservation and analytical procedures in the Materials and Methods section.

Changes made: Materials and Methods (Lines 152–160):

Line 95-96: References are needed to support the claim of inconsistent patterns across ecosystems.

Response: We thank the reviewer for this helpful comment. We agree that this statement should be supported by appropriate references. Accordingly, we have added representative references to support the observation that the assembly patterns of core and non-core microbial taxa vary among different ecosystems. These references provide additional context for our statement and strengthen the rationale for investigating this question in marine surface waters.

Changes made: Introduction (Lines 99–104).

Line 107: The transition using 'In contrast' may not be entirely smooth in this context.

Response: We thank the reviewer for this helpful suggestion. We have revised the manuscript accordingly and replaced "In contrast" with "However" to improve the logical flow and readability of the text.

Changes made: Introduction (Line 114).

Line 142: The sample pre-treatment procedure for physicochemical analyses is not sufficiently described.

Response: We thank the reviewer for this helpful comment. We have revised the manuscript by adding a more detailed description of the sample pre-treatment procedures for physicochemical analyses. Specifically, we now clarify the sample volume, the filtration procedure for dissolved nutrient analyses, the storage conditions,

and the different pre-treatment protocols for total nitrogen and total phosphorus measurements, as well as the analytical instrument used. We believe these additions improve the transparency and reproducibility of the methodology.

Changes made: Materials and Methods (Line 152–160).

Line 170-173: The authors cite references for defining core taxa that are based on OTU-level analyses, not ASVs. Since ASVs resolve single-nucleotide variants and contain more rare sequences than OTUs, it is unclear whether OTU-based thresholds are appropriate. The authors should clarify or justify their approach.

Response: We thank the reviewer for this insightful comment. We agree that ASVs provide higher taxonomic resolution than OTUs and can resolve additional rare sequence variants. However, in our study, core taxa were defined using stringent occurrence and abundance criteria, with core taxa required to be detected in all samples and to have a relative abundance $\geq 0.2\%$ in more than 80% of samples. Because ASVs uniquely resolved by ASV-based methods are predominantly rare and infrequently detected, they are unlikely to substantially affect the identification of core taxa under these criteria. To further clarify and justify our approach, we have revised the manuscript to cite both the original studies establishing the occurrence-based concept of core taxa using OTUs and more recent studies applying the same framework to ASV-based datasets. These revisions clarify that the concept is applicable across different sequence resolution strategies while acknowledging the higher taxonomic resolution provided by ASVs.

Changes made: Materials and Methods (Lines 187).

Line 209-210: Please check the supplementary table citation, as Supplementary Table S3 should likely be Supplementary Table S1.

Response: We thank the reviewer for pointing out this error. We have corrected the manuscript and ensured that the geographic coordinates are now correctly referred to in Supplementary Table 1 instead of Supplementary Table 3. We have also carefully checked the manuscript to ensure that no similar referencing errors remain.

Changes made: Materials and Methods (Lines 228–229).

Line 224: across THE sampling sites.

Response: We thank the reviewer for carefully identifying this minor issue. We have added the missing “the” to correct the phrase to “across the sampling sites.”

Changes made: Results (Line 238).

Line 234 and elsewhere: change 367 {plus minus} 14 to 367{plus minus}14. No space should be left between the numbers and {plus minus}. The same applies to similar circumstances throughout the manuscript.

Response: We thank the reviewer for this helpful comment. We have carefully revised the manuscript and removed the spaces around the “±” symbol throughout to ensure consistent formatting.

Changes made: Manuscript (throughout).

Line 241: In general, the presentation of results should be more specific and quantitative. Whenever possible, descriptive terms such as "highest", "lowest", "largest proportion", or "dominant" should be supported by the corresponding numerical values.

Response: We thank the reviewer for this helpful suggestion. We agree that providing quantitative values alongside descriptive terms improves the clarity and precision of the Results. Accordingly, we have revised the relevant text by adding the corresponding numerical values to support descriptions such as "highest", "lowest", "largest proportion", and "dominant" wherever appropriate.

Changes made: Results (Lines 254–260).

Line 247-253: The placement of the network analysis results under the section "Composition and diversity of microbial communities with contrasting core and non-core taxa" seems inappropriate. Consider creating a separate subsection for network analysis or modifying the section title accordingly.

Response: We thank the reviewer for this helpful suggestion. We agree that the original subsection title did not fully reflect the content presented, particularly the inclusion of the co-occurrence network analysis. Accordingly, we have revised the subsection title

to **"Composition, diversity, and co-occurrence network characteristics of core and non-core microbial taxa"**, which more accurately represents the scope of the results and improves the organization and readability of the Results section.

Changes made: Results (Lines 239–240).

Line 254-255: This subsection is relatively long (over one page) and contains extensive detail. It may benefit from being streamlined to emphasize the key results.

Response: We thank the reviewer for this helpful suggestion. We agree that this subsection was relatively detailed and could benefit from being streamlined. Accordingly, we have revised the text by reducing redundant descriptions and removing some less essential numerical details, while retaining the key quantitative results and main patterns for each analysis. We believe these revisions improve the conciseness and readability of this section without affecting the interpretation of the results.

Changes made: Results (Lines 270–310).

Line 277-283 and 324: Similar to the comment on line 241.

Response: We thank the reviewer for this helpful comment. We agree that numerical information should be used appropriately to support descriptive interpretations. For the random forest analysis, variable importance is primarily interpreted based on ranking; therefore, we have retained the original presentation while ensuring that the ranking information of key variables across different community types is clearly and consistently described.

Changes made: Results (Lines 285–298; 328–341).

Line 322-336: The main finding concerns contrasting assembly processes between core and non-core communities, yet the environmental correlation analyses are performed on the overall community and do not directly address these group-specific patterns. I would be more interested in identifying which environmental factors drive the assembly of non-core taxa, and it may be more appropriate to present the corresponding figures in the main text rather than in the supplementary materials.

Response: We thank the reviewer for this insightful suggestion. We agree that identifying the environmental factors associated with the assembly processes of core and non-core communities would further strengthen the comparison between these two groups. We therefore carefully evaluated the feasibility of conducting separate analyses. However, this was not possible for the core community because the assembly metric (|RC|) showed essentially no variation among pairwise comparisons. Consequently, the response variable required for random forest analysis was effectively constant, making a separate model for the core community statistically infeasible. Consequently, a separate random forest analysis of environmental variables associated with the assembly processes of the core community could not be performed in a statistically meaningful manner.

Regarding the reviewer's suggestion to move the corresponding figures into the main text, we appreciate this recommendation. However, because these analyses were conducted only for the whole community rather than separately for the core and non-core communities, we believe that presenting them in the Supplementary Materials better maintains the focus of the main text on the contrasting assembly patterns between core and non-core taxa, while still providing the complete analytical results for interested readers.

Line 337-364: The functional analysis is conducted at the whole-community level, which may not align with the manuscript's focus on core versus non-core taxa. Consider analyzing functional structure separately for core and non-core communities to maintain consistency with the study's main theme.

Response: We thank the reviewer for this helpful comment. We agree that distinguishing between core and non-core taxa is central to the study. However, the functional predictions in this study were generated using PICRUSt2 based on 16S rRNA gene data, which provides community-level estimates of functional potential rather than reliable taxon-resolved functional profiles. Therefore, separating functional analyses into core and non-core fractions would introduce additional uncertainty due to reduced sequence representation within each subset. For this reason, we conducted the functional analysis at the whole-community level.

Figures

General problem: The font sizes in the figures are generally too small. Please increase the font size of labels, legends, and annotations to ensure readability.

Response: We thank the reviewer for this helpful suggestion. We agree that improving figure readability is important. Accordingly, we have increased the font sizes of labels, legends, and annotations in all figures to improve clarity and readability.

Changes made: Font sizes have been increased throughout all figures in the revised manuscript.

Fig. 1: I love the fancy, colourful figures, but they do not server your purposes. Your point is that there is little variation among the sampling sites, but those Ocean Viewer figures usually show spatial variations. In addition, the font sizes of the physicochemical parameter labels are not consistent. Please unify the formatting.

Response: We thank the reviewer for this helpful comment. We agree that consistency in figure formatting is important and have unified the font sizes and label styles in Fig. 1 accordingly to improve clarity.

Regarding the purpose of Fig. 1, this figure was designed to provide an overview of the sampling locations and the spatial distribution of key physicochemical parameters, thereby complementing the detailed numerical data presented in the Supplementary Tables and Results section. We have ensured that the presentation now better emphasizes the relatively limited variation among sampling sites, while retaining its role in illustrating the geographic context of the study.

Changes made: Figure. 1 has been revised to unify font sizes and improve visual consistency.

Fig. 2: what are the numbers on the x-axis for the panels e and f.

Response: We thank the reviewer for this helpful comment. We agree that the x-axis labels in Fig. 2e–f were not sufficiently clear in the original version. We have revised the figure by explicitly labeling the x-axis to indicate the number of ASVs for both core and non-core taxa to improve clarity.

Changes made: Figure. 2e–f has been revised to clarify that the x-axis represents the number of ASVs.

Data Availability

The sequencing data are not available yet.

Response: We thank the reviewer for this comment. We would like to clarify that the sequencing data have already been deposited in the NCBI Sequence Read Archive under Bioproject PRJNA1460329. This information has been clearly provided in the "Availability of Data and Materials" section (Lines 539–541). We have further ensured that the data availability statement is clearly presented in the revised manuscript.

Reviewer #5 (Comments for the Author):

General Comments

This study investigated the surface-water microbial communities in the Xisha Islands, a marine environment characterized by low environmental heterogeneity, to explore the relative impacts of environmental and spatial factors on the community assembly mechanisms and ecological functional potentials of core and non-core taxa. However, from the perspective of rigorous academic evaluation and the publication standards of AEM, this manuscript exhibits fundamental deficiencies in its formulation of scientific questions, experimental design logic, and the objective robustness of its conclusions.

First, the manuscript lacks a well-defined core scientific hypothesis and a rigorous experimental design. In essence, this study is a regional descriptive and survey-based investigation that relies heavily on localized spatial sampling. Consequently, it falls short in terms of mechanistic depth and theoretical generalizability. Second, although this study place a heavy emphasis on biogeochemical cycling functions, the actual study completely lacks experimental validation targeting specific elemental cycles. Instead, it relies solely on static functional predictions via PICRUSt2 based on 16S rRNA amplicon sequencing data. Drawing definitive conclusions regarding complex and dynamic ecological assembly mechanisms and biogeochemical implications from such highly limited and speculative predictive profiling is inherently unrigorous and lacks an objective foundation. The conventional bioinformatic analyses and superficial data streams presented here cannot robustly support the ecological conclusions the authors attempt to articulate.

Overall, while this study holds certain value as a regional survey, the scientific questions are unfocused, and the work lacks a clear hypothesis-driven approach and mechanistic verification. The current dataset and analytical framework are insufficient to substantiate the primary ecological conclusions proposed. The findings are more suitable for publication as a regional microbial diversity survey or a descriptive community study, rather than meeting the requirements of a high-impact microbial ecology journal centered on ecological mechanisms or biogeochemical processes. Therefore, the authors are encouraged to re-conceptualize their scientific questions and supplement their work with lines of evidence at the functional and process levels. If

these missing components cannot be provided, the authors should consider resubmitting the manuscript to a journal focused primarily on regional surveys and community descriptions.

Response: We thank the reviewer for the comprehensive evaluation of our manuscript and for raising important concerns regarding the scientific framing, analytical interpretation, and scope of our conclusions. We acknowledge that this study is based on field observations from a regional marine system and does not aim to experimentally validate universal mechanisms or directly quantify biogeochemical processes. However, we would like to clarify that the primary objective of this study is not merely to describe microbial diversity in the Xisha Islands, but to address a broader ecological question regarding how environmental and spatial processes contribute to marine microbial community organization, particularly for taxa exhibiting contrasting ecological distributions and occurrence patterns.

Environmental and spatial factors are widely recognized as important components shaping marine microbial communities, yet their relative contributions remain incompletely understood, especially in marine environments characterized by relatively low environmental heterogeneity. The Xisha Islands therefore provide a valuable regional case study to investigate whether subtle environmental gradients and spatial processes are associated with differences in community assembly patterns between core and non-core taxa.

In response to the reviewer's concerns, we have revised the manuscript to better define the scope of our study and avoid overgeneralization of our findings. Specifically, we have strengthened the interpretation of environmental and spatial effects by incorporating variation partitioning analysis and have revised the terminology throughout the manuscript to distinguish statistical associations from causal mechanisms.

Regarding functional analyses, we agree that PICRUSt2-based inference cannot replace direct measurements of functional genes, transcripts, or biogeochemical processes. Accordingly, we have revised the manuscript to consistently describe these results as predicted functional potential rather than measured microbial functions. We have also added discussion regarding the limitations of functional prediction and removed

statements that could imply direct evidence for ecosystem functions or elemental cycling processes.

We believe that these revisions have improved the conceptual clarity, analytical rigor, and appropriate interpretation of our findings, while maintaining the focus of this study on microbial community assembly patterns in a tropical marine ecosystem.

Specific Comments

L170: The authors defined core taxa as those "present in all samples with a relative abundance greater than 0.2% in more than 80% of the samples." However, the selection of this specific threshold lacks a clear ecological justification. With only 42 ASVs classified as core taxa under this definition, the conclusions of this study might be highly sensitive to the chosen threshold. The authors should further clarify the rationale behind this criterion and conduct a sensitivity analysis using alternative occupancy and abundance thresholds to verify the robustness and universality of their primary conclusions.

Response: We thank the reviewer for this thoughtful comment. We agree that there is currently no universally accepted criterion for defining core taxa, and different studies have adopted different combinations of occupancy and abundance thresholds depending on their research objectives. The criterion used in this study follows previously published approaches and the corresponding references have been added in the revised manuscript.

The primary objective of our study was not to establish or optimize a definition of the core microbiome, but rather to compare the ecological characteristics and assembly patterns of core and non-core taxa under a consistent classification framework. Our results demonstrate that distinguishing core from non-core taxa reveals contrasting ecological patterns that are not apparent when the community is analyzed as a whole. Although alternative thresholds may change the number of ASVs assigned to each category, we believe that this does not alter the conceptual framework or the main ecological interpretations of the present study.

L381: The manuscript repeatedly emphasizes that the physicochemical conditions in

the studied region varied within a very narrow range, yet subsequently highlights temperature and pH as the most critical environmental drivers shaping community structure and function. Whether a statistically "significant correlation" under such narrow fluctuations possesses any real physiological and ecological significance for microorganisms is highly questionable. The authors need to clarify whether such marginal variations in temperature and pH are sufficient to exert strong environmental selection pressures, or if the observed variations in the microbial community are instead driven by unmeasured oceanographic processes, with temperature and pH merely serving as collinear proxies for these complex physicochemical dynamics.

Response: We thank the reviewer for this insightful comment. We agree that, in a marine environment characterized by relatively low environmental heterogeneity, statistically significant relationships do not necessarily imply direct physiological selection or causal environmental effects. Accordingly, we have revised the manuscript to avoid overinterpreting the measured physicochemical variables as direct environmental drivers and instead describe them as variables associated with microbial community composition and predicted functional profiles.

We also acknowledge that the measured physicochemical variables may reflect broader oceanographic processes or covary with unmeasured environmental factors. Therefore, the observed relationships should be interpreted as statistical associations rather than direct evidence of causal environmental selection. To address this point, we have added a corresponding statement to the Discussion to clarify this limitation and provide a more cautious interpretation of the environmental associations.

Changes made: Discussion (Lines 422–426).

L447: The conclusion that the assembly of core taxa is "governed exclusively by dispersal limitation" lacks ecological plausibility. As widely distributed and dominant members of the community, core taxa typically possess strong dispersal capabilities or a broad adaptation to regional environmental conditions, rather than exhibiting characteristics of a typically dispersal-limited group. Furthermore, correlation analyses indicated that environmental variables are significantly associated with the core community composition, suggesting that environmental filtering still plays an important role. Therefore, the "100% dispersal limitation" result stands in stark

contradiction to both the ecological attributes of the core taxa and other analytical findings in this paper. The authors must further validate the robustness of this conclusion and discuss whether it is an artifact of the core taxa definition, the small sample size, or the inherent limitations of the null model approach itself.

Response: We thank the reviewer for this thoughtful comment. We agree that the interpretation of the null-model results requires careful consideration. In the revised manuscript, we have moderated the discussion of the assembly processes and clarified that the inferred association with dispersal limitation should not be interpreted as evidence that core taxa are dispersal-limited in terms of their occurrence across sampling sites. Instead, because core taxa were present in all samples, the null-model results more likely reflect spatially structured variation in their relative abundances rather than differences in taxon occurrence.

We also note that the null-model analysis and the environmental correlation analyses address different ecological questions. While the null model infers the dominant community assembly processes based on phylogenetic turnover, the correlation analyses and variation partitioning analysis evaluate statistical associations between community variation and measured environmental and spatial variables. Accordingly, significant environmental associations are not inconsistent with the null-model results. To further strengthen this interpretation, we incorporated variation partitioning analysis into the revised manuscript and expanded the Discussion to clarify the different ecological implications of these complementary analytical approaches.

Regarding the possibility that the observed pattern may be influenced by the definition of core taxa or the limitations of the null-model framework, we acknowledge these as potential considerations and have discussed the interpretation of the null-model results more cautiously in the revised manuscript.

Changes made: Discussion (Lines 474–477); Figure 3g–i; Materials and Methods (Lines 206–210); Results (Lines 303–307); Discussion (Lines 478–482) and Abstract (Lines 35–39).

Re: Spectrum02178-26R1 (**Divergent assembly patterns of core and non-core microbial taxa under low environmental heterogeneity in the surface waters of the Xisha Islands**)

Dear Mr. Zhixuan Deng:

Your manuscript has been accepted, and I am forwarding it to the ASM production staff for publication. Your paper will first be checked to make sure all elements meet the technical requirements. ASM staff will contact you if anything needs to be revised before copyediting and production can begin. Otherwise, you will be notified when your proofs are ready to be viewed.

Data Availability: ASM policy requires that data be available to the public upon online posting of the article, so please verify all links to sequence records, if present, and make sure that each number retrieves the full record of the data. If a new accession number is not linked or a link is broken, provide production staff with the correct URL for the record. If the accession numbers for new data are not publicly accessible before the expected online posting of the article, publication may be delayed; please contact ASM production staff immediately with the expected release date.

Publication Fees: For information on publication fees and which article types have charges, please visit ourwebsite. We have partnered with Copyright Clearance Center (CCC) to collect author charges. If fees apply to your paper, you will receive a message from no-reply@copyright.com with further instructions. For questions related to paying charges through RightsLink, please contact CCC at ASM_Support@copyright.com or toll free at +1-877-622-5543. CCC makes every attempt to respond to all emails within 24 hours.

ASM Membership: Corresponding authors may join or renew ASM membership to obtain discounts on publication fees. Need to upgrade your membership level? Please contact Customer Service at Service@asmusa.org.

Embargo Policy: A press release may be issued as soon as the manuscript is posted on theSpectrum Latest Articles webpage. The corresponding author will receive an email with the subject line "ASM Journals Author Services Notification" when the article is available online.

Cover Image Submissions: If you would like to submit a potential Cover Image, please email a file and a short legend to spectrum@asmusa.org. Please note that we can only consider images that (i) the authors created or own and (ii) have not been previously published. By submitting, you agree that the image can be used under the same terms as the published article. Image File requirements: TIF/EPS, 7.5 inches wide by 8.25 inches tall (at least 2,250 pixels wide by 2,475 pixels tall), minimum 300 dpi resolution (600 dpi preferred), RGB, and no figure elements, e.g., arrows or panel labels. The legend should be a short description of the image, 1-2 sentences recommended. Please download and use this interactive template in Adobe the https://journals.asm.org/pb-assets/html/asm-cover-sizing-tool.html to ensure that your proposed cover image meets our size requirements (https://journals.asm.org/pb-assets/pdf-text-excel-files/ASM-Interactive-Sizing-Cover-Template-1715689791.pdf).

The ASM Journals program strives for constant improvement in our submission and publication process. Please tell us how we can improve your experience by taking this quick Author Survey.

Thank you for submitting your paper to Spectrum.

Sincerely,
Jan Claesen
Editor
Microbiology Spectrum
