# Supplementary material for: Divergent assembly patterns of core and non-core microbial taxa under low environmental heterogeneity in the surface waters of the Xisha Islands
Source: Microbiol Spectr. 2026 Aug 14;14(9):e02178-26. doi: 10.1128/spectrum.02178-26 (PMC13532294; doi:10.1128/spectrum.02178-26)
Supplement: Supplementary file 2 [file spectrum.02178-26-s0005.docx]

**Figure** **captions**

**Figure 1. Environmental and geographic characteristics of surface waters in the Xisha Islands.** Sampling sites were distributed across the regional surface waters of the Xisha Islands, representing the spatial extent of the study area. Physicochemical parameters of the surface waters of the Xisha Islands. The heatmap showing the variation in environmental parameters, with colors representing their values.

**Figure 2.** **Composition and diversity of microbial communities in the surface waters of the Xisha Islands.** (a) The unique and shared ASVs among the 13 sampling sites. (b) The ten most abundant bacterial phyla in water. (c) Percentage of core and non-core taxa. (d) Relative abundance of core and non-core taxa. (e) Taxonomic composition of core taxa at the phylum level. (f) Taxonomic composition of non-core taxa at the phylum level. (g) Co-occurrence network analysis of microbial relationships. The network is based on pairwise Spearman’s correlations; red edges represent positive correlations, and blue edges represent negative correlations. Node size indicates the degree. Red nodes represent core ASVs, and gray nodes represent non-core ASVs.

**Figure 3. Environmental and geographic drivers of microbial community.** Random forest analysis showing the variable importance of environmental variables and geographic distance in predicting Bray-Curtis community dissimilarity for the (a) whole community, (b) core taxa, and (c) non-core taxa. Variable importance is expressed as the percentage increase in mean squared error (%IncMSE), with larger values indicating a greater contribution to model performance. Distance-decay curves of similarity for the (d) whole community, (e) core taxa, and (f) non-core taxa. The red line represents the fitted linear regression with R² and *P* values labeled. VPA of the effects of geographic factors and environmental factors the (g) whole community, (h) core taxa, and (i) non-core taxa bacterial structure. *** *P* < 0.01.

**Figure 4. The contribution of ecological processes on the microbial assembly.** Ecological processes of the (a) whole community, (b) core taxa, and (c) non-core taxa.

**Figure 5. Relative abundances of microbial community functional genes and their relationships with environmental factors.** Relative abundances of genes in （a）level 2 categories and （b） level 3 categories. (c) Correlations between the relative abundances of genes and environmental parameters. The colour gradient on the right represents the Spearman’s rank correlation coefficients, with more positive values (dark red) indicating stronger positive correlations and more negative values (dark blue) indicating stronger negative correlations. Asterisks denote significance levels: *** *P* < 0.001, ** *P* < 0.01, * *P* < 0.05.

**Supplementary figure 1. Composition of microbial communities in the surface waters of the Xisha Islands.** The ten most abundant bacterial genus in water.

**Supplementary figure 2. Environmental and geographic correlates of microbial alpha diversity and composition.** (a) Correlations between the microbial alpha diversity and environmental parameters. Number of (b) core ASVs and (c) non-core ASVs significantly correlated with each environmental parameter. (d) Correlations between the relative abundances of core ASVs and environmental parameters. (e) Correlations between the relative abundances of non-core ASVs and environmental parameters. The colour gradient on the right represents the Spearman’s rank correlation coefficients, with more positive values (dark red) indicating stronger positive correlations and more negative values (dark blue) indicating stronger negative correlations. Asterisks denote the significance levels of the Spearman’s rank correlation coefficients. *** *P* < 0.001, ** *P* < 0.01, * *P* < 0.05.

**Supplementary figure 3. Environmental drivers of microbial community assembly.** Random forest analysis showing the variable importance scores (%IncMSE) of environmental variables associated with variation in microbial community assembly processes.

**Supplementary figure 4. Geographic drivers of microbial community assembly.** Distance–decay curves showing the similarity for the relationship between geographic distance and |RC| across sample pairs.

**Table captions**

**Supplementary table 1 Environmental and geographic characteristics of surface waters in the Xisha Islands.**

Geographical coordinates, temperature (T, °C), pH, dissolved oxygen (DO, mg L⁻¹), oxidation-reduction potential (ORP, mV), salinity (SAL, ppt), ammonium (NH₄⁺-N, mg L⁻¹), nitrite (NO₂⁻-N, mg L⁻¹), nitrate (NO₃⁻-N, mg L⁻¹), phosphate (PO₄³⁻-P, mg L⁻¹), total nitrogen (TN, mg L⁻¹), and total phosphorus (TP, mg L⁻¹) of the surface water at each sampling site.

**Supplementary table 2 Summary statistics of environmental variables measured in the surface waters of the Xisha Islands.** Values are presented as mean ± standard deviation (SD), observed range (minimum–maximum), and coefficient of variation (CV) across the sampling sites. Temperature (T, °C), pH, dissolved oxygen (DO, mg L⁻¹), oxidation-reduction potential (ORP, mV), salinity (SAL, ppt), ammonium (NH₄⁺-N, mg L⁻¹), nitrite (NO₂⁻-N, mg L⁻¹), nitrate (NO₃⁻-N, mg L⁻¹), phosphate (PO₄³⁻-P, mg L⁻¹), total nitrogen (TN, mg L⁻¹), and total phosphorus (TP, mg L⁻¹).

**Supplementary table 3 Alpha diversity of the microbial community.**

Alpha diversity indices were calculated based on the ASV table.

**Supplementary table 4 Topological information of the microbial community co-occurrence network.**

The topological analysis of the symbiotic network of microorganisms was carried out using R software and Gephi software.

**Supplementary table 5 Correlations between geographical/environmental factors and whole microbial community composition.**

The Mantel test was based on Bray–Curtis dissimilarity. *r*, correlation coefficient; *P*, significance level. Asterisks indicate significant correlations: *** *P* < 0.001, ** *P* < 0.01, * *P* < 0.05. “All” represents the combined effect of all geographical and environmental factors.

**Supplementary table 6 Correlations between geographical/environmental factors and microbial community composition of the core taxa.**

The Mantel test was based on Bray–Curtis dissimilarity. *r*, correlation coefficient; *P*, significance level. Asterisks indicate significant correlations: *** *P* < 0.001, ** *P* < 0.01, * *P* < 0.05. “All” represents the combined effect of all geographical and environmental factors.

**Supplementary table 7 Correlations between geographical/environmental factors and microbial community composition of the non-core taxa.**

The Mantel test was based on Bray–Curtis dissimilarity. *r*, correlation coefficient; *P*, significance level. Asterisks indicate significant correlations: *** *P* < 0.001, ** *P* < 0.01, * *P* < 0.05. “All” represents the combined effect of all geographical and environmental factors.

**Supplementary table 8 Importance of environmental variables associated with microbial community assembly.**

Random forest analysis showing the relative importance of environmental parameter variables in explaining the variation in microbial community assembly processes. Variable importance was quantified using the percentage increase in mean squared error (%IncMSE), where higher values indicate a stronger contribution to model perform. IncNodePurity, increase in node purity, representing the total reduction in node impurity due to splits on the variable. Higher values indicate stronger importance.

**Supplementary table 9 Correlations between geographical distance and microbial community assembly.**

The Spearman correlation coefficient analysis was based on |RC| of microbial community. *r*, correlation coefficient; *P*, significance level. Asterisks indicate significant correlations: *** *P* < 0.001, ** *P* < 0.01, * *P* < 0.05.
